# Dietary Docosahexaenoic Acid Prevents Silica-Induced Development of Pulmonary Ectopic Germinal Centers and Glomerulonephritis in the Lupus-Prone NZBWF1 Mouse

**DOI:** 10.3389/fimmu.2018.02002

**Published:** 2018-09-12

**Authors:** Melissa A. Bates, Peyman Akbari, Kristen N. Gilley, James G. Wagner, Ning Li, Anna K. Kopec, Kathryn A. Wierenga, Daven Jackson-Humbles, Christina Brandenberger, Andrij Holian, Abby D. Benninghoff, Jack R. Harkema, James J. Pestka

**Affiliations:** ^1^Department of Food Science and Human Nutrition, Michigan State University, East Lansing, MI, United States; ^2^Institute for Integrative Toxicology, Michigan State University, East Lansing, MI, United States; ^3^Department of Pathobiology and Diagnostic Investigation, Michigan State University, East Lansing, MI, United States; ^4^Department of Biochemistry and Molecular Biology, Michigan State University, East Lansing, MI, United States; ^5^Institute of Functional and Applied Anatomy, Hannover Medical School, Hannover, Germany; ^6^Department of Biomedical and Pharmaceutical Sciences, Center for Environmental Health Sciences, University of Montana, Missoula, MT, United States; ^7^Department of Animal, Dairy and Veterinary Sciences, School of Veterinary Medicine, Utah State University, Logan, UT, United States; ^8^Department of Microbiology and Molecular Genetics, Michigan State University, East Lansing, MI, United States

**Keywords:** autoimmunity, ectopic lymphoid structure, omega-3, polyunsaturated fatty acid, systemic lupus erythematosus, silica, lung

## Abstract

Ectopic lymphoid structures (ELS) consist of B-cell and T-cell aggregates that are initiated *de novo* in inflamed tissues outside of secondary lymphoid organs. When organized within follicular dendritic cell (FDC) networks, ELS contain functional germinal centers that can yield autoantibody-secreting plasma cells and promote autoimmune disease. Intranasal instillation of lupus-prone mice with crystalline silica (cSiO_2_), a respirable particle linked to human lupus, triggers ELS formation in the lung, systemic autoantibodies, and early onset of glomerulonephritis. Here we tested the hypothesis that consumption of docosahexaenoic acid (DHA), an ω-3 polyunsaturated fatty acid with anti-inflammatory properties, influences the temporal profile of cSiO_2_-induced pulmonary ectopic germinal center formation and development of glomerulonephritis. Female NZBWF1 mice (6-wk old) were fed purified isocaloric diets supplemented with 0, 4, or 10 g/kg DHA - calorically equivalent to 0, 2, or 5 g DHA per day consumption by humans, respectively. Beginning at age 8 wk, mice were intranasally instilled with 1 mg cSiO_2_, or saline vehicle alone, once per wk, for 4 wk. Cohorts were sacrificed 1, 5, 9, or 13 wk post-instillation (PI) of the last cSiO_2_ dose, and lung and kidney lesions were investigated by histopathology. Tissue fatty acid analyses confirmed uniform dose-dependent DHA incorporation across all cohorts. As early as 1 wk PI, inflammation comprising of B (CD45R^+^) and T (CD3^+^) cell accumulation was observed in lungs of cSiO_2_-treated mice compared to vehicle controls; these responses intensified over time. Marked follicular dendritic cell (FDC; CD21^+^/CD35^+^) networking appeared at 9 and 13 wk PI. IgG^+^ plasma cells suggestive of mature germinal centers were evident at 13 wk. DHA supplementation dramatically suppressed cSiO_2_-triggered B-cell, T-cell, FDC, and IgG^+^ plasma cell appearance in the lungs as well as anti-dsDNA IgG in bronchial lavage fluid and plasma over the course of the experiment. cSiO_2_ induced glomerulonephritis with concomitant B-cell accumulation in the renal cortex at 13 wk PI but this response was abrogated by DHA feeding. Taken together, realistic dietary DHA supplementation prevented initiation and/or progression of ectopic lymphoid neogenesis, germinal center development, systemic autoantibody elevation, and resultant glomerulonephritis in this unique preclinical model of environment-triggered lupus.

## Introduction

Systemic lupus erythematosus (lupus) is a heterogeneous autoimmune disease (AD) that primarily affects young women causing great individual suffering and social costs (1, 2). Induction and progression of this disease is coordinated by aberrant activation of innate and adaptive immunity. Pathogenesis involves loss of tolerance to self-antigens, activation of autoreactive B- and T-cells and consequent production of pathogenic autoantibodies (3). The latter complex with self-antigens such as dsDNA, forming circulating immune complexes that amass within tissues. In the kidney, these complexes drive mononuclear cell infiltration and activation, promoting glomerulonephritis that often culminates in end-stage renal disease (4).

During unresolved inflammation, ectopic lymphoid structures (ELS) are induced to remediate high concentrations of locally-present antigens in inflamed tissue and, thus, do not occur in pre-programmed locations in the body. Also known as tertiary lymphoid organs, ELS operate much like secondary lymphoid organs; they serve structurally and functionally as a site for antigen accumulation, presentation, and proliferation/activation of B- and T-cells. B- and T-cell-rich areas are distinct with the former being supported within a matrix of follicular dendritic cells (FDCs) (5–7). Unlike hematopoietic myeloid and plasmacytoid dendritic cells, FDCs are considered mesenchymal in nature. They express low levels of major histocompatibility complex (MHC) II and are identified by their surface expression of FcγRIIb, complement receptor (CR) 1 (CD35), and CR2 (CD21) [reviewed in (8)]. These receptors facilitate FDC presentation of immune complexes of antibody or complement-opsonized antigens. FDCs dictate the fate of B-cells in germinal centers by serving as a platform for their recruiting, activation, differentiation, and survival (9). Importantly, ELS are frequently associated with lupus and other ADs reflecting the intimate association between aberrant inflammation and loss of self-tolerance. ELS are found in AD target tissues such as kidneys (lupus), salivary glands (Sjögren's syndrome), and synovial joints (rheumatoid arthritis) and correlate with disease severity (6, 10–14).

Although lupus and other ADs are driven by an individual's genome, environmental factors such as toxicants and diet can influence onset and severity of autoimmunity (15–20). Regarding toxicants, it is well-established that inhaled crystalline silica dust (cSiO_2_, quartz) can contribute to AD onset and progression [reviewed in (21)]. Approximately 2.3 million individuals who work in occupations such as mining, construction, manufacturing, and farming are exposed to respirable cSiO_2_ (22). Airway exposure to cSiO_2_ evokes overt and chronic inflammation that affects not only the lung, but also distal organs by way of systemic inflammation (23, 24). Epidemiological studies have linked cSiO_2_ exposure to lupus, rheumatoid arthritis, Sjögren's syndrome, scleroderma, and systemic vasculitis (25–29).

Animal studies provide mechanistic insight into how cSiO_2_ might trigger autoimmunity in humans (30–33). Intranasal cSiO_2_ instillation of the female NZBWF1 mouse, a widely used murine model of human lupus (34), induces autoimmunity and glomerulonephritis 3 months earlier than vehicle-instilled controls (35). Concurrently, there are massive inflammatory responses in the lungs as evidenced by intense perivascular and peribronchial lymphocytic cell infiltration. These infiltrates contain many B (CD45R^+^) cells and T (CD3^+^) cells indicative of ectopic lymphoid neogenesis. Consistent with pulmonary inflammation and ELS formation, bronchoalveolar lavage fluid (BALF) from cSiO_2_-exposed mice contain elevated concentrations of IgG, autoantibodies, chemokines, and cytokines. These responses are similarly reflected in the plasma and, therefore, indicative of parallel exacerbation of systemic autoimmunity. Overall, these findings strongly indicate that the lung acts as a platform for autoimmune triggering by cSiO_2_.

Dietary intake of polyunsaturated fatty acids (PUFAs) may have considerable influence on inflammation and autoimmunity [reviewed in (36)]. PUFAs that have two or more double bonds are designated as ω-3 or ω-6 based on the position of the first double bond relative to the aliphatic chain's terminal carbon. Generally, ω-3 PUFAs are recognized to be anti-inflammatory in nature, whereas ω-6 PUFAs are regarded as proinflammatory (37). Linoleic acid (C18:2 ω-6; LA), the most common PUFA in the Western diet, is present in food oils produced from plants such as corn and soybeans. After consumption by ruminants and humans, LA can be elongated and desaturated enzymatically to arachidonic acid (C20:4 ω-6; ARA). Thus, LA and ARA are major components of cell membrane phospholipids. Even though the ω-3 PUFA α-linolenic acid (C18:3 ω-3; ALA) is additionally present in plant oils, humans and other mammals have limited ability to elongate it enzymatically to more anti-inflammatory forms, docosahexaenoic acid (C22:6 ω-3; DHA) and eicosapentaenoic acid (C20:5 ω-3; EPA). Therefore, humans need to obtain these PUFAs from exogenous sources. Several marine algae proficiently catalyze formation of DHA and EPA, and cold-water fish consuming these algae efficiently bioconcentrate these PUFAs into their tissues (38). Accordingly, fish, fish oil, and microalgal oil are significant sources of DHA and EPA in the human diet (39).

Numerous preclinical investigations have demonstrated that ω-3 PUFA-rich fish oil supplementation both prevents and resolves inflammation [reviewed in (40, 41)]. Therefore, its consumption might also benefit people with a genetic predisposition to AD. In support of this contention, fish oil supplementation of lupus-prone mice prevents or delays glomerulonephritis and death from kidney failure, with DHA-enriched fish oil having the greatest potency (42–50). In a prior work, we compared latency and severity of autoimmunity in female NZBWF1 mice consuming (1) an ω-3 PUFA-rich diet containing DHA-enriched fish oil, (2) an ω-6 PUFA-rich Western-type diet containing corn oil, or (3) an ω-9 monounsaturated fatty acid (MUFA)-rich Mediterranean-type diet containing high oleic safflower oil (51). While increases of plasma autoantibodies, proteinuria, and glomerulonephritis were readily evident in mice consuming ω-6 PUFA or ω-9 MUFA diets, these outcomes were remarkably lessened in animals fed the DHA-rich diet. These inhibitory effects correlated with downregulation of genes associated with inflammatory responses, antigen presentation, T-cell activation, B-cell activation/differentiation and leukocyte recruitment. Importantly, a large number of these genes are specific targets for lupus intervention.

Recently, we queried how DHA supplementation influences cSiO_2_-induced autoimmunity in female NZBWF1 mice. Dietary DHA at concentrations that were calorically equivalent to 2, 6, and 12 g per day human consumption dose-dependently suppressed cSiO_2_-triggered inflammation, accumulation of pulmonary B- and T-cell aggregates, autoimmunity, and glomerulonephritis (52). While these observations offer the intriguing possibility that DHA supplementation could be used to block environment-triggered lupus, such an interpretation is constrained because the investigation focused only on a single time point−3 months following the final cSiO_2_ instillation and after glomerulonephritis onset. Furthermore, the presence of B- and T-cell aggregates was not explicitly linked to the presence of FDCs and plasma cells. To address these knowledge gaps, in the present study we determined how dietary DHA supplementation, at two concentrations that realistically mimic safe human consumption (2 and 5 g per day), influences the temporal profile of cSiO_2_-triggered (1) pulmonary B-cells, T-cells, FDCs, and plasma cells indicative of ectopic germinal centers, (2) systemic autoimmunity, and (3) glomerulonephritis in the female NZBWF1 lupus model.

## Materials and methods

### Animals and diets

All experimental protocols were reviewed and approved by the Institutional Animal Care and Use Committee at Michigan State University in accordance with the National Institutes of Health guidelines (AUF #01/15-021-00). Female 6-wk-old lupus-prone NZBWF1 mice were obtained from The Jackson Laboratories (Bar Harbor, ME), housed four per cage with free access to food and water, and kept at constant temperature (21–24°C) and humidity (40–55%) under a 12 h light/dark cycle.

Upon arrival and for the duration of the study, mice were fed one of four experimental diets (Table 1). Formulations were based on the semi-purified American Institute of Nutrition (AIN)-93G containing 70 g/kg fat (53). To provide basal essential fatty acids (FAs), all diets contained 10 g/kg corn oil. Control diet (CON) was formulated with 60 g/kg high-oleic safflower oil (Hain Pure Food, Boulder, CO). To prepare DHA-enriched diets, high-oleic safflower oil was substituted with 10 g/kg (Low DHA) or 25 g/kg (High DHA) microalgal oil containing 40% DHA (DHASCO, kindly provided by Dr. Kevin Hadley, DSM Nutritional Products, Columbia MD). This yielded experimental diets that contained 4 or 10 g/kg DHA, respectively, and modeled, on a caloric basis, human DHA consumption of 2 and 5 g per day, respectively.

**Table 1:** Diet formulations.

	**CON**	**Low DHA^e^**	**High DHA^f^**
Casein	200	200	200
Dyetrose	132	132	132
Cornstarch	397.5	397.5	397.5
Sucrose	100	100	100
Cellulose	50	50	50
t-Butylhydroquinone (TBHQ)	0.01	0.01	0.01
AIN 93G Salt Mix	35	35	35
AIN 93G Vitamin Mix (with Vitamin E)	10	10	10
L-cysteine	3	3	3
Choline Bitartrate	2.5	2.5	2.5
Corn Oil^a^^,^ ^b^	10	10	10
High-oleic safflower Oil^a^^,^ ^c^	60	50	35
DHA-Enriched Algal Oil^a^^,^ ^d^	–	10	25

*Values shown are the mass amounts (g) per kg diet*.

a *Based on oil composition reported by the manufacturer*.

b *Corn oil contained 612 g/kg linoleic acid and 26 g/kg oleic acid*.

c *High-oleic safflower oil contained 750 g/kg oleic acid and 140 g/kg linoleic acid*.

d *Algal oil contained 395 g DHA/kg and 215 g oleic acid/kg*.

e *4 g/kg diet, calorically equivalent to human DHA consumption of 2 g/day*.

f *10 g/kg diet, calorically equivalent to human DHA consumption of 5 g/day*.

### Experimental design

**Figure 1:**
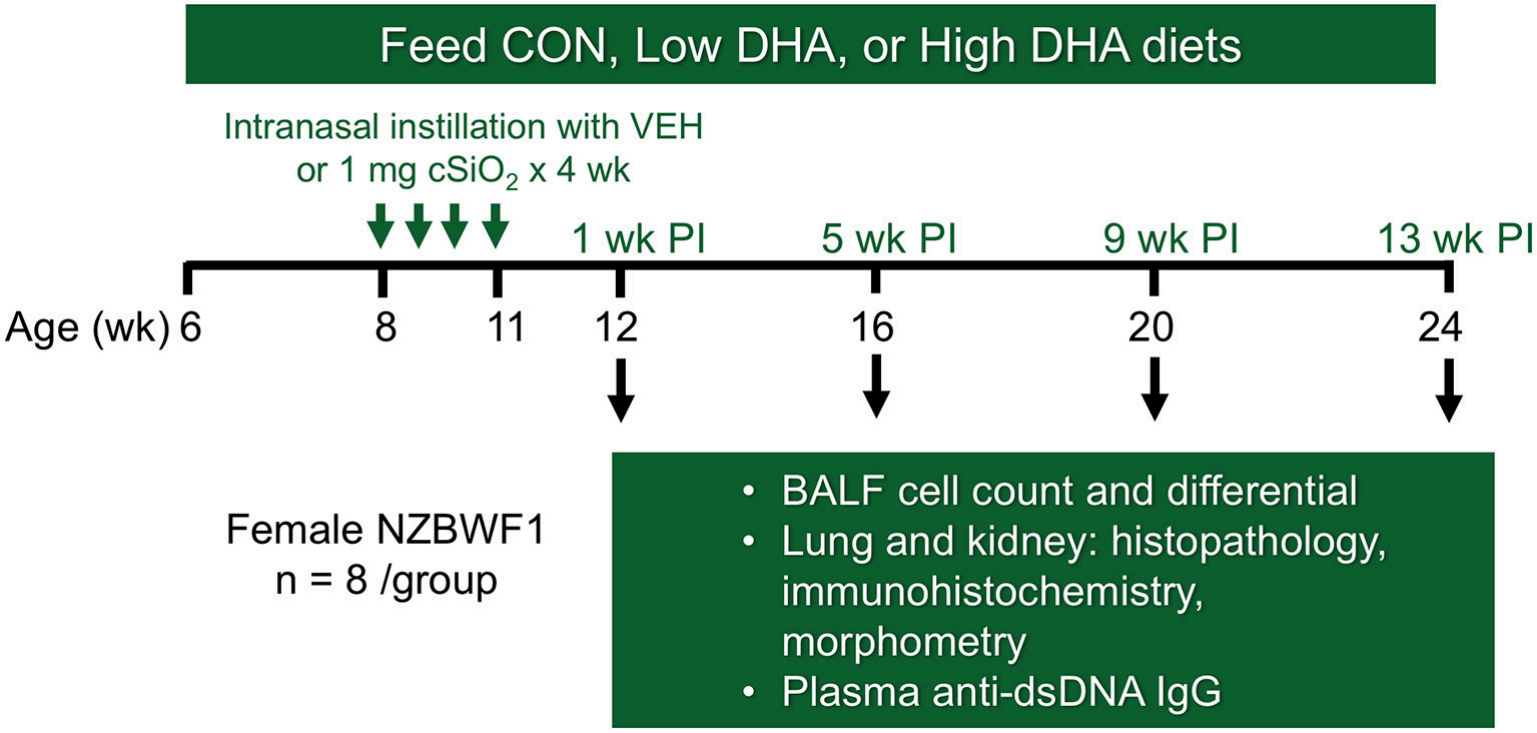
Experimental design. Feeding of groups of female NZBWF1 mice with CON (0 g/kg DHA), Low DHA (4 g/kg DHA) or High DHA (10 g/kg DHA) diets were initiated at age 6 wk. At age 8 wk, mice were dosed intranasally with 25 μl PBS VEH or 25 μl PBS containing 1 mg cSiO_2_ weekly for 4 wk. Cohorts (*n* = 7–8 per group) of animals were terminated at 12, 16, 20, and 24 wk of age which corresponded to 1, 5, 9, and 13 wk post-instillation (PI) of final cSiO_2_ dose.

The experimental design for this study is depicted in Figure 1. At 6 wk of age, groups of mice (*n* = 8) were fed CON, Low DHA, or High DHA diets and maintained on assigned diets until the end of the experiment. To prevent the formation of lipid oxidation products, diets were mixed weekly and stored at −20°C until use. Fresh feed was provided ad lib to mice daily. At 8 wk of age, mice were anesthetized with 4% isoflurane and intranasally instilled with 1 mg cSiO_2_ in 25 μl phosphate buffered saline (PBS) or the PBS vehicle (VEH). Exposures were repeated once per wk for 4 wk. cSiO_2_ (Min-U-Sil-5, 1.5–2.0 μm average particle size, Pennsylvania Sand Glass Corporation, Pittsburgh, PA) was acid washed and dried prior to preparation of stock suspensions of cSiO_2_ in VEH. Stock suspensions were prepared fresh in VEH prior to exposure, sonicated, and vortexed rapidly for 1 min prior to intranasal instillation. This dosing regimen reflects approximately one half of a human lifetime exposure at the recommended Occupational Safety and Health Administration limit (35).

Cohorts of mice representing the four treatment groups (i.e., CON/VEH, CON/cSiO_2_, Low DHA/cSiO_2_, and High DHA/cSiO_2_) were sacrificed at 1, 5, 9, and 13 wk after the final cSiO_2_ instillation. Mice were 12, 16, 20, and 24 wk of age, respectively, at these time points. These time periods were selected to capture pulmonary pathological changes in lupus-prone NZBWF1 mice following cSiO_2_ exposure prior to and during onset of glomerulonephritis based on prior studies (35, 52). Animals were euthanized by intraperitoneal injection with 56 mg/kg body weight sodium pentobarbital and exsanguination via the abdominal aorta. Blood was collected with heparin-coated syringes and centrifuged at 3500 × g for 10 min at 4°C for separation of erythrocytes and plasma which were stored at −80°C. BALF was collected from whole lungs as described previously (54). After lavage, the right lung lobes were removed, frozen in liquid nitrogen, and stored at −80°C for FDC immunohistochemistry. The left lung lobe was fixed with 10% neutral buffered formalin (Fisher Scientific, Pittsburgh, PA) at constant pressure (30 cm H_2_O) for minimum of 1 h and stored in fixative until further processing for histology. The right kidney was excised and the cranial portion fixed in 10% neutral buffered formalin for 24 h.

### Fatty acid analyses

Fatty acid (FA) concentrations in diets were determined by gas liquid chromatography (GLC) as previously described (52). FA data are reported as percentages (w/w) of FAs detected with a chain length between 10 and 24 carbon atoms. The lower limit of detection was < 0.001 g/100 g fatty acids. FA concentrations in erythrocytes were analyzed by GLC at Omega Quant (Sioux Falls, SD).

### BALF cell counts

Total cells in BALF were determined by counting on a hemocytometer. Cytological slides were prepared by centrifugation of 150 μl BALF for 10 min at 400 × g, air-drying, and staining with Diff-Quick (Thermo-Fisher). Two hundred (200) cells were counted and designated as monocytes/macrophages, lymphocytes, and eosinophils using morphological criteria.

### Anti-dsDNA IgG analysis

Anti-dsDNA IgG responses in BALF and plasma were measured using mouse anti-dsDNA IgG-specific ELISA Kit (Alpha Diagnostic International, San Antonio, TX) according to manufacturer's instructions. Briefly, samples as well as standards were added to plate wells, incubated and bound antibody was measured using horseradish peroxidase (HRP)-labeled anti-mouse IgG. Anti-dsDNA in samples was determined from standard curve generated with mouse anti-dsDNA.

### Histopathology of lungs and kidneys

Randomly oriented, serial sections of formalin-fixed left lung lobes were processed routinely and embedded in paraffin. Tissue sections (5 μm) were deparaffinized and stained with hematoxylin and eosin (H&E) for histopathology. Tissues were scored semi-quantitatively in a blinded fashion by a board-certified veterinary pathologist for the following lung lesions: (a) presence of lymphoid aggregates within perivascular and peribronchiolar regions; (b) histologically evident ectopic lymphoid tissues; (c) presence of alveolar proteinosis; (d) alveolitis defined as the increased accumulation in the alveolar parenchyma of neutrophils, lymphocytes, and mononuclear/macrophages; (e) alveolar type II epithelial cell hyperplasia; and (f) mucous cell metaplasia in bronchiolar epithelium. Individual lungs were graded for these lesions using the following criteria (% of total pulmonary tissue examined): (0) no changes compared to control mice; (1) minimal (<10%); (2) slight (10–25%); (3) moderate (26–50%); (4) severe (51–75%); or (5) very severe (>75%) of total area affected.

Formalin-fixed kidneys were randomly sectioned, paraffin-embedded, cut and stained with either H&E or Periodic acid-Schiff and hematoxylin (PASH). Stained sections were evaluated for lupus nephritis by a board-certified veterinary pathologist using a modified International Society of Nephrology/Renal Pathology (ISN/RPS) Lupus Nephritis Classification system (55). Slide sections were graded as follows: (0) no tubular proteinosis and normal glomeruli; (1) mild tubular proteinosis with multifocal segmental proliferative glomerulonephritis and occasional early glomerular sclerosis and crescent formation; (2) moderate tubular proteinosis with diffuse segmental proliferative glomerulonephritis, early glomerular sclerosis and crescent formation; and (3) marked tubular proteinosis with diffuse global proliferative and sclerosing glomerulonephritis. The extent of tubular necrosis was measured in PASH-stained slides by morphometry as described below.

### Immunohistochemistry of lungs and kidneys

Immunohistochemistry was performed on formalin-fixed, paraffin embedded, left lung lobe and right kidney for identification of CD45R^+^ (B-cells). Sections were cut at 7 μm on a microtome and placed on slides coated with 2% 3-aminopropyltriethoxysilane and dried at 56°C overnight. Slides were deparaffinized in xylene and rehydrated through descending grades of ethyl alcohol to distilled water followed by Tris Buffered Saline (TBS) (pH 7.4) without surfactant (Scytek Labs, Logan, UT) for 5 min for pH adjustment. Following TBS treatment, paraffin-embedded tissue sections underwent heat induced epitope recovery with Citrate Plus Retrieval pH 6.0 (Scytek Labs) for 30 mins at 125°C followed by a 30 min incubation at room temperature (RT). Endogenous peroxidase was blocked with 3% hydrogen peroxide and methanol (1:4) for 30 min followed by tap and distilled water rinses.

After pretreatments, standard micro-polymer complex staining was performed at RT on an IntelliPath Flex Autostainer (Biocare Medical, Pacheco, CA). All staining steps were followed by rinses in TBS autowash buffer (Biocare). After blocking for non-specific protein with Rodent Block M (Biocare) for 20 min, sections were incubated with 1:600 rat anti-CD45R monoclonal antibody (Becton Dickinson, Franklin Lakes, NJ; catalog # 550286) in normal antibody diluent (Scytek) for 1 h to identify B-cells. Bound CD45R antibody was detected with ProMark Rat-on-Mouse HRP Polymer by 30 min incubations with probe and polymer, respectively, and reaction developed with Romulin 3-amino-9-ethylcarbazole (AEC) chromogen (Scytek) prior to being counterstained with 1:10 hematoxylin for 1 min followed by distilled water. Slides were air dried and cover slips then applied with permanent synthetic mounting media.

For identification of CD3^+^ (T-cells) in lung parenchyma, following deparaffinization and rehydration, tissue sections underwent heat induced epitope retrieval with Citrate Plus Retrieval pH 6.0 (Scytek) for 30 min at 100°C followed by a 10 min incubation at RT. Endogenous peroxidase was blocked as described above, and non-specific binding was blocked with Rodent Block M for 10 min. After pretreatments, 1:250 rabbit anti-CD3 polyclonal antibody (Abcam, Cambridge, MA; catalog #ab5690) was incubated for 1 h to identify T-cells. Bound CD3 antibody was detected with ProMark Rat-on- Mouse HRP polymer (Biocare) with 15 min incubation for probe and polymer, respectively and reaction developed with AEC for 5 min prior to being counterstained with hematoxylin.

For detection of IgG^+^ cells in lung, deparaffinized sections were incubated with 0.03% pronase E for 10 min at 37°C to achieve antigen retrieval and then endogenous peroxidase inactivated as described above. After blocking for non-specific protein with Rodent Block M (Biocare) sections were incubated with 1:100 polyclonal goat anti-IgG (Bethyl Labs, Montgomery, TX; catalog# A-90-100A) for 30 min. Goat-specific Micro-Polymer (Biocare) reagents were subsequently applied as per manufacturer's instructions, followed by reaction development with Romulin AEC™ (Biocare) and counterstained with hematoxylin.

Immunohistochemistry for CD21^+^/CD35^+^ (FDC) was performed on snap frozen cranial lobe of the right lung. Tissues were sectioned at 7 μm and sections air dried overnight on 2% 3-aminopropyltriethoxysilane coated slides. Sections were fixed with acetate and formalin for 10 min at RT, and endogenous peroxidase was blocked using 3% hydrogen peroxide in TBS for 30 min. Blocking for non-specific binding was performed with Rodent Block M (Biocare) for 5 min. Follow pretreatments, 1:500 rat anti-CD21/35 monoclonal antibody (Becton Dickinson; catalog # 553817) was incubated for 1 h to identify FDCs. Bound CD21/CD35 antibody was detected with ProMark Rat-on-Mouse HRP polymer (Biocare) with 15 min incubation for probe and polymer, respectively and reaction developed with AEC for 5 min prior to being counterstained with hematoxylin.

### Morphometry

To quantitate immunohistochemically labeled cells in lungs (CD45R^+^, CD3^+^, and CD21^+^/CD35^+^) and kidneys (CD45R^+^) indicative of ELS, tissue morphometry was performed as described previously (52). Briefly, slides were digitized with a VS110 Virtual Slide System (Olympus). The entire tissue was selected as the region of interest and 20% of the sections were captured by systematic random sampling with NewCast software (Visiopharm, Hoersholm, Denmark) and a virtual magnification of 20X. Percentages of CD45R^+^, CD3^+^, or CD21/CD35^+^ cells were calculated by projecting a point grid over randomly sampled images with the STEPanizer v 1.8 stereology tool (56) and tallying the number of points falling onto positive staining or reference tissue.

### Statistics

All treatments consisted of 8 mice per group, and data are presented as mean ± SEM. One mouse in the 5 wk CON/VEH group died of unknown causes during the course of the experiment; it did not show the typical signs of sickness including loss of body weight before death. The Shapiro-Wilk test was used to determine if data were distributed normally, and the robust outlier (ROUT) test (*Q* = 1%) was used to identify putative outliers. For data sets that met assumptions for normality and equal variance, a two-way ANOVA was used with treatment and time point as the experimental factors. For data that were normally distributed but unequal in variance, a log_10_ transformation was applied and the parametric two-way ANOVA was performed. Then, for each time point, the Sidak's *post-hoc* test for multiple comparisons was used for the following pairwise comparisons identified *a priori*: CON/VEH vs. CON/cSiO_2_; CON/cSiO_2_ vs. Low DHA/cSiO_2_; and CON/cSiO_2_ vs. High DHA/cSiO_2_. For data obtained at a single time point, a one-way ANOVA was performed with the same *post-hoc* pairwise comparisons. Because the vast majority of histopathology score data were not normally distributed and not suitable for typical data transformations, these data were analyzed using the nonparametric Kruskal-Wallis ANOVA on ranks (either two-way or one-way, depending on the data set) followed by Dunn's multiple comparisons tests for the pairwise tests outlined above. A *p* value < 0.05 was considered statistically different for main effects of treatment, time point or treatment x time point and for the pairwise comparisons to the negative (CON/VEH) or positive control (CON/cSiO_2_) for all study outcomes. Statistical analyses were performed using GraphPad Prism v.7 (La Jolla, CA).

## Results

### DHA is incorporated in tissues at the expense of ARA

GLC analysis confirmed that projected FA content of experimental diets (Table 1) was consistent with their final compositions (Table 2). Erythrocytes were further analyzed to ascertain how experimental diets influenced tissue FA profiles (57). Consumption of DHA (C22:6 ω-3) resulted in increased erythrocyte concentrations of this FA and concurrently decreased ARA (C20:4 ω-6) at 1 wk PI (Table 3). Interestingly, although DHA was the predominant ω-3 PUFA, EPA (C20:5 ω-3) accounted for 16 and 21 percent of total ω-PUFAs in erythrocytes from mice fed the Low DHA and High DHA diets, respectively. FA content in erythrocytes at 1 wk PI (Table 3) did not appreciably differ from that of the 5, 9, or 13 wk PI (Supplementary Tables 1–3). This suggested that ω-3 PUFA incorporation peaked within 6 wk of initiating DHA feeding and remained stable throughout the course of the experiment.

**Table 2:** Fatty acid (FA) content of experimental diets.

	**CON**	**Low DHA**	**High DHA**
C12:0	0.04 ± 0.00	0.62 ± 0.00	1.42 ± 0.07
C14:0	0.16 ± 0.01	1.79 ± 0.02	4.15 ± 0.21
C15:0	0.03 ± 0.00	0.02 ± 0.00	0.03 ± 0.00
C16:0	5.64 ± 0.04	6.82 ± 0.02	7.79 ± 0.02
C16:1 7 c/8 c	0.03 ± 0.00	0.03 ± 0.00	0.03 ± 0.00
C16:1 9 c	0.08 ± 0.00	0.41 ± 0.00	0.86 ± 0.03
C17:0	0.03 ± 0.00	0.04 ± 0.00	0.04 ± 0.00
C18:0	1.71 ± 0.01	1.56 ± 0.01	1.39 ± 0.02
C18:1 9 c	71.08 ± 0.13	62.40 ± 0.08	53.26 ± 0.50
C18:1 11 c	0.75 ± 0.02	0.65 ± 0.01	0.55 ± 0.01
C18:2 (ω-6)	19.17 ± 0.10	18.94 ± 0.01	15.08 ± 0.79
C20:0	0.31 ± 0.00	0.28 ± 0.00	0.24 ± 0.00
C18:3 (ω-3)	0.36 ± 0.01	0.37 ± 0.00	0.32 ± 0.01
C22:0	0.22 ± 0.00	0.21 ± 0.00	0.20 ± 0.00
C24:0	0.14 ± 0.00	0.13 ± 0.00	0.12 ± 0.01
C22:5 (ω-3)	0.00 ± 0.00	0.09 ± 0.00	0.20 ± 0.01
C22:6 (ω-3)	0.00 ± 0.00	5.40 ± 0.05^a^	14.20 ± 0.65^b^
∑ SFA	8.3 ± 0.1	11.5 ± 0.1	15.4 ± 0.1
∑ MUFA	71.9 ± 0.1	63.5 ± 0.1	54.7 ± 0.5
∑PUFA (ω-3)	0.4 ± 0.01	5.9 ± 0.1	14.7 ± 0.7
∑ PUFA (ω-6)	19.2 ± 0.1	18.9 ± 0.0	15.1 ± 0.8
ω-6:ω-3	52.8 ± 1.3	3.2 ± 0.0	1.0 ± 0.1

*Data shown are mean percent of total lipid in diet ± SEM or the ratio of ω-6 to ω-3 FAs of three representative diets as determined by GLC*.

a *Equivalent to 3.9 g/kg DHA diet*.

b *Equivalent to 9.9 g/kg DHA diet*.

**Table 3:** Fatty acid (FA) content of erythrocytes at 1 wk post-instillation (PI).

	**CON/VEH**	**CON/cSiO_2_**	**Low DHA/cSiO_2_**	**High DHA/cSiO_2_**
C14:0	0.15 ± 0.01	0.15 ± 0.01	0.23 ± 0.03	0.29 ± 0.03
C16:0	24.79 ± 0.43	24.70 ± 0.45	27.13 ± 0.54	29.11 ± 0.48
C16:1n7t	0.04 ± 0.01	0.04 ± 0.01	0.03 ± 0.01	0.03 ± 0.01
C16:1n7	0.59 ± 0.08	0.64 ± 0.03	0.61 ± 0.61	0.63 ± 0.10
C18:0	16.04 ± 0.42	15.87 ± 0.22	15.07 ± 0.32	14.30 ± 0.48
C18:1t	0.16 ± 0.01	0.16 ± 0.01	0.15 ± 0.01	0.14 ± 0.01
C18:1 ω-9	17.28 ± 0.51	17.19 ± 0.26	15.77 ± 0.54	15.01 ± 0.14
C18:2 ω-6t	0.06 ± 0.01	0.07 ± 0.01	0.05 ± 0.00	0.05 ± 0.00
C18:2 ω-6	9.35 ± 0.058	9.24 ± 0.51	11.71 ± 0.45	11.15 ± 0.75
C20:0	0.16 ± 0.02	0.14 ± 0.02	0.16 ± 0.02	0.15 ± 0.01
C18:3 ω-6	0.06 ± 0.02	0.07 ± 0.01	0.05 ± 0.01	0.03 ± 0.00
C20:1n9	0.43 ± 0.02	0.39 ± 0.02	0.30 ± 0.03	0.23 ± 0.02
C18:3 ω-3	0.04 ± 0.00	0.04 ± 0.00	0.05 ± 0.01	0.04 ± 0.01
C20:2 ω-6	0.27 ± 0.02	0.27 ± 0.01	0.25 ± 0.03	0.17 ± 0.01
C22:0	0.12 ± 0.01	0.11 ± 0.03	0.13 ± 0.03	0.13 ± 0.02
C20:3 ω-6	1.36 ± 0.10	1.43 ± 0.07	1.35 ± 0.16	0.80 ± 0.06
C20:4 ω-6	19.56 ± 0.89	19.97 ± 0.56	8.46 ± 0.50	3.90 ± 0.22
C24:0	0.21 ± 0.03	0.22 ± 0.05	0.25 ± 0.05	0.29 ± 0.05
C20:5 ω-3	0.24 ± 0.04	0.30 ± 0.03	2.75 ± 0.33	4.80 ± 0.26
C24:1 ω-9	0.24 ± 0.04	0.23 ± 0.06	0.24 ± 0.05	0.23 ± 0.04
C22:4 ω-6	1.98 ± 0.15	1.88 ± 0.06	0.38 ± 0.04	0.16 ± 0.02
C22:5 ω-6	0.68 ± 0.04	0.65 ± 0.04	0.09 ± 0.02	0.05 ± 0.01
C22:5 ω-3	0.58 ± 0.05	0.61 ± 0.03	0.94 ± 0.04	0.91 ± 0.05
C22:6 ω-3	5.59 ± 0.36	5.62 ± 0.20	13.85 ± 0.42	17.40 ± 0.64
∑ SFA	41.47 ± 0.49	41.19 ± 0.44	42.97 ± 0.46	44.26 ± 0.53
∑ MUFA	18.74 ± 0.49	18.66 ± 0.26	17.10 ± 0.63	16.27 ± 0.15
∑ PUFA (ω-3)	6.45 ± 0.44	6.57 ± 0.21	17.58 ± 0.58	23.15 ± 0.84
∑ PUFA (ω-6)	33.34 ± 0.51	33.58 ± 0.41	22.35 ± 0.84	16.32 ± 0.82

*Analysis of experimental groups from 1 wk after final cSiO_2_ instillation. Data shown are mean percent of total FA ± SEM (n = 8 per group) as determined by GLC. Data for 5, 9, and 13 wk PI can be found in Supplementary Tables 1–3, respectively*.

### DHA consumption suppresses cSiO_2_-induced mononuclear cell elevation in BALF

**Figure 2:**
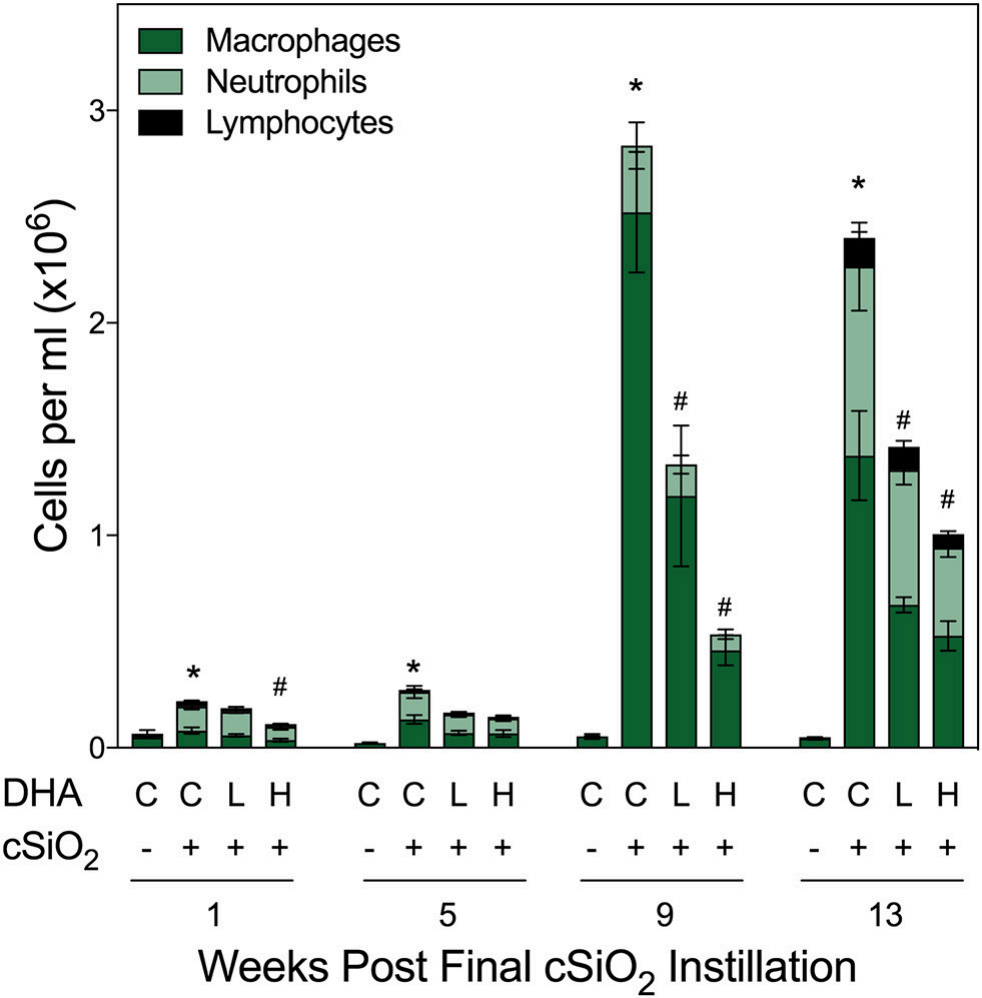
DHA supplementation suppresses cSiO_2_-induced elevation of inflammatory cells in BALF. Total cell counts in BALF samples from NZBWF1 mice were conducted using a hemocytometer and then monocytes/macrophages, lymphocytes, neutrophils, and eosinophils quantified after differential staining using morphological criteria. Abbreviations: C, control diet; L, Low DHA diet; H, High DHA diet. Data are means ± SEM (*n* = 7–8 per group). Symbols: ^*^ indicates significantly different from CON/VEH group (*p* < 0.05); # indicates significantly different from CON/cSiO_2_ group (*p* < 0.05). Complete statistical analyses can be found in Supplementary Table 5.

Total cell counts in BALF of cSiO_2_-exposed mice fed CON diet were modestly increased at 1 and 5 wk PI compared to VEH-treated mice fed CON diet (Figure 2). By 9 and 13 wk PI, cell numbers in the CON/cSiO_2_ group were dramatically elevated. Rank order of the inflammatory cell populations was macrophage > neutrophils > lymphocytes with eosinophils being negligibly affected. cSiO_2_-induced inflammatory cell recruitment in the lung alveolar space was suppressed significantly at 9 and 13 wk PI in mice consuming the Low DHA and High DHA diets (Figure 2, Supplementary Table 5).

### DHA supplementation blocks cSiO_2_-induced inflammation and cell infiltration in lung

**Figure 3:**
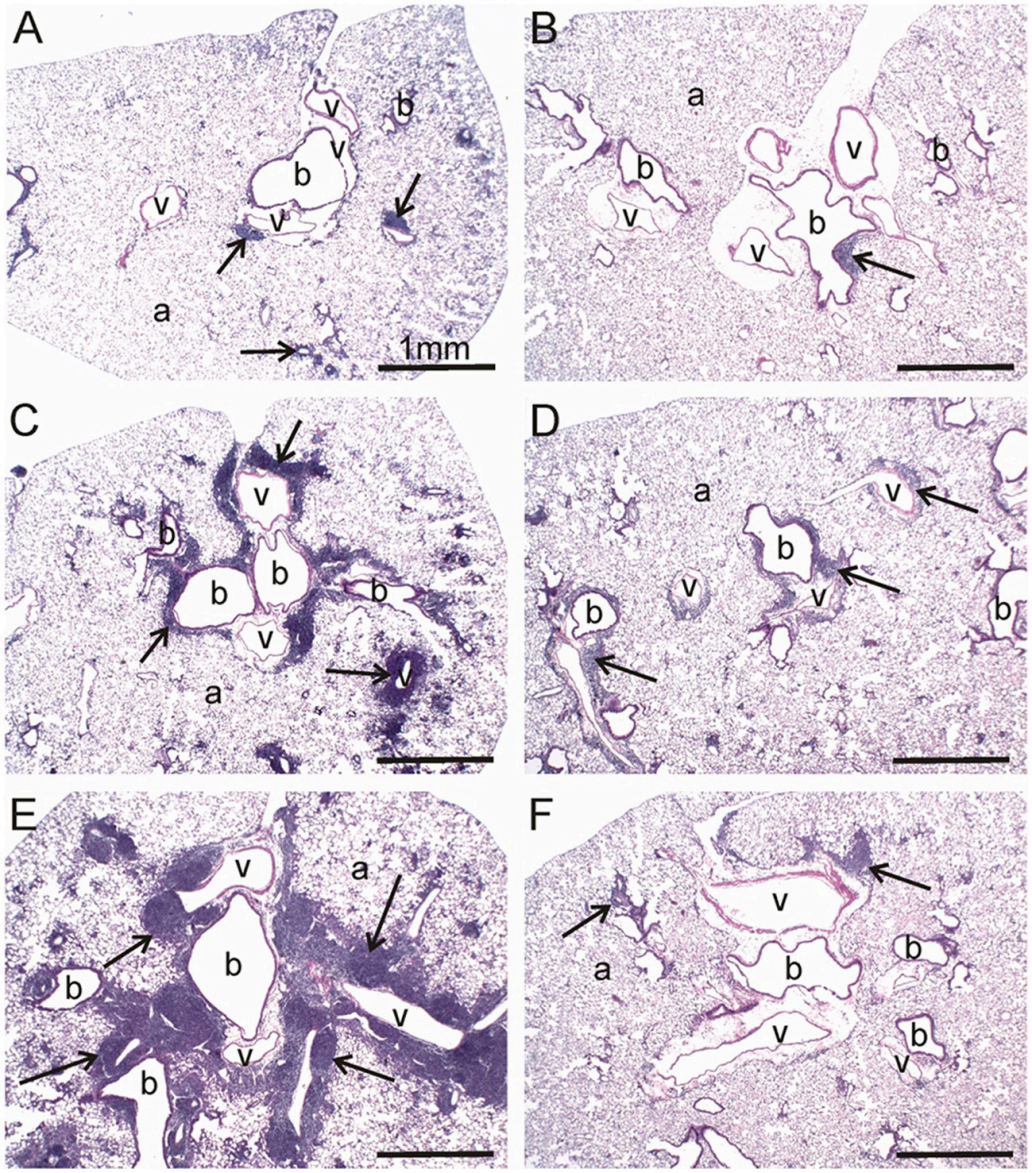
DHA consumption prevents cSiO_2_-triggered inflammation in lungs of NZBWF1 mice. Representative light photomicrographs depict H&E-stained lung sections from CON/cSiO_2_ groups at **(A)** 5 wk post-instillation (PI), **(C)** 9 wk PI, and **(E)** 13 wk PI; **(B)** CON/VEH at 13 wk PI; **(D)** Low DHA/cSiO_2_ at 13 wk PI; and **(F)** High DHA/cSiO_2_ at 13 wk PI. Black arrows in photomicrographs indicate lymphoid cell infiltration in the peribronchiolar and perivasculature interstitium. Dietary DHA substantially suppressed lymphocytic infiltration (**D**, **F**; See also Table 4). Abbreviations: a, alveolar parenchyma; b, bronchiolar airway; v, blood vessel.

At 1 and 5 wk PI, small lesions were evident in the lungs of the CON/cSiO_2_ group as compared to CON/VEH group (Figure 3A, Table 4). Lymphoid aggregates in interstitial tissue circumventing the airways and blood vessels were present in some cSiO_2_-treated mice; mild alveolar proteinosis and alveolitis were similarly apparent. By 9 wk PI, pulmonary lesions in the CON/cSiO_2_ group were more severe (Figure 3C, Table 4, Supplementary Table 4). ELS formation closely paralleled increases in lymphoid aggregates in CON/cSiO_2_ mice. These features were predominately present as interstitial lymphocytic infiltrates encompassing the airways and blood vessels in the lungs. Consumption of the High DHA diet diminished the size of these lymphocytic lesions at 9 wk PI but did not appreciably impact development of alveolar proteinosis or type II alveolar epithelial cell hyperplasia.

**Table 4:** Histopathological assessment of cSiO_2_-triggered pulmonary inflammation in female NZBWF1 mice fed CON or DHA-enriched diets at 1, 5, 9, and 13 wk post-instillation.

	**Week**	**CON/VEH**	**CON/cSiO_2_**	**Low DHA/cSiO_2_**	**High DHA/cSiO_2_**
Lymphoid aggregates	1	0.6 ± 0.2	1.1 ± 0.1	0.9 ± 0.1	0.8 ± 0.2
	5	0.6 ± 0.2	1.5 ± 0.2^*^	1.1 ± 0.1	0.8 ± 0.1
	9	1.0 ± 0.0	3.1 ± 0.2^*^	2.2 ± 0.1	1.1 ± 0.2^#^
	13	0.9 ± 0.1	3.6 ± 0.2^*^	2.0 ± 0.2^#^	1.6 ± 0.2^#^
Ectopic lymphoid structures	1	0.0 ± 0.0	0.2 ± 0.2	0.0 ± 0.0	0.0 ± 0.0
	5	0.0 ± 0.0	0.1 ± 0.1	0.0 ± 0.0	0.0 ± 0.0
	9	0.4 ± 0.0	2.4 ± 0.2^*^	1.2 ± 0.2	0.6 ± 0.2^#^
	13	0.0 ± 0.0	3.4 ± 0.2^*^	1.6 ± 0.3	1.1 ± 0.3^#^
Alveolar proteinosis	1	0.0 ± 0.0	1.5 ± 0.3^*^	1.1 ± 0.1	1.0 ± 0.0
	5	0.0 ± 0.0	2.2 ± 0.2^*^	2.2 ± 0.2	2.1 ± 0.1
	9	0.0 ± 0.0	4.0 ± 0.2^*^	4.0 ± 0.0	4.0 ± 0.0
	13	0.0 ± 0.0	4.0 ± 0.1^*^	4.1 ± 0.1	4.1 ± 0.1
Alveolitis	1	0.0 ± 0.0	1.0 ± 0.0^*^	1.0 ± 0.0	0.4 ± 0.1^#^
	5	0.0 ± 0.0	1.2 ± 0.2^*^	1.0 ± 0.0	1.0 ± 0.0
	9	0.0 ± 0.0	2.6 ± 0.3^*^	1.9 ± 0.2	1.1 ± 0.2^#^
	13	0.0 ± 0.0	2.8 ± 0.2^*^	2.2 ± 0.2	2.0 ± 0.2
Type II alveolar epithelial cell hyperplasia	1	0.0 ± 0.0	0.0 ± 0.0	0.0 ± 0.0	0.0 ± 0.0
	5	0.0 ± 0.0	0.0 ± 0.0	0.0 ± 0.0	0.0 ± 0.0
	9	0.0 ± 0.0	0.6 ± 0.2^*^	0.6 ± 0.2	0.6 ± 0.2
	13	0.0 ± 0.0	2.2 ± 0.2^*^	1.6 ± 0.3	1.6 ± 0.3
Mucous cell metaplasia	1	0.0 ± 0.0	0.0 ± 0.0	0.0 ± 0.0	0.0 ± 0.0
	5	0.0 ± 0.0	0.0 ± 0.0	0.0 ± 0.0	0.0 ± 0.0
	9	0.0 ± 0.0	0.0 ± 0.0	0.0 ± 0.0	0.0 ± 0.0
	13	0.0 ± 0.0	2.2 ± 0.2	0.4 ± 0.2	0.1 ± 0.1

*NZBWF1 mice (n = 7–8/group) were graded individually for severity of lung inflammation (% of total pulmonary tissue) as follows: 0, no changes; 1, minimal (<10%); 2, slight (10–25%); 3, moderate (26–50%); 4, severe (51–75%); 5, very severe (>75%) of total area affected. Data are shown as mean ± SEM score for each pathological lesion*.

* *indicates treatment group is significantly different compared to CON/VEH (p < 0.05)*.

# *indicates treatment group is significantly different compared to CON/cSiO_2_ (p < 0.05). Complete statistical results are provided in Supplementary Table 4*.

Consistent with previous findings (35, 52), cSiO_2_ triggered severe inflammation and ELS expansion at 13 wk PI (Figures 3B,E, Table 4). In addition, mucous cell metaplasia in the respiratory epithelium lining large-diameter bronchioles was observed that was not evident at earlier time points. Attenuation of lymphocyte-dependent lesions (i.e., lymphoid aggregates and ELS) was evident in both the Low DHA and High DHA groups (Figures 3D,F, Table 4, Supplementary Table 4). Consumption of the High DHA diet reduced cSiO_2_-induced alveolitis at this time point, but this response was not evident in mice fed the Low DHA diet. However, DHA did not markedly impact cSiO_2_-induced lymphocyte-independent histopathological lesions including alveolar proteinosis, type II alveolar epithelial cell hyperplasia, and mucous cell metaplasia.

### Dietary DHA delays cSiO_2_-induced accumulation of B- and T-cell aggregates in lung over time

**Figure 4:**
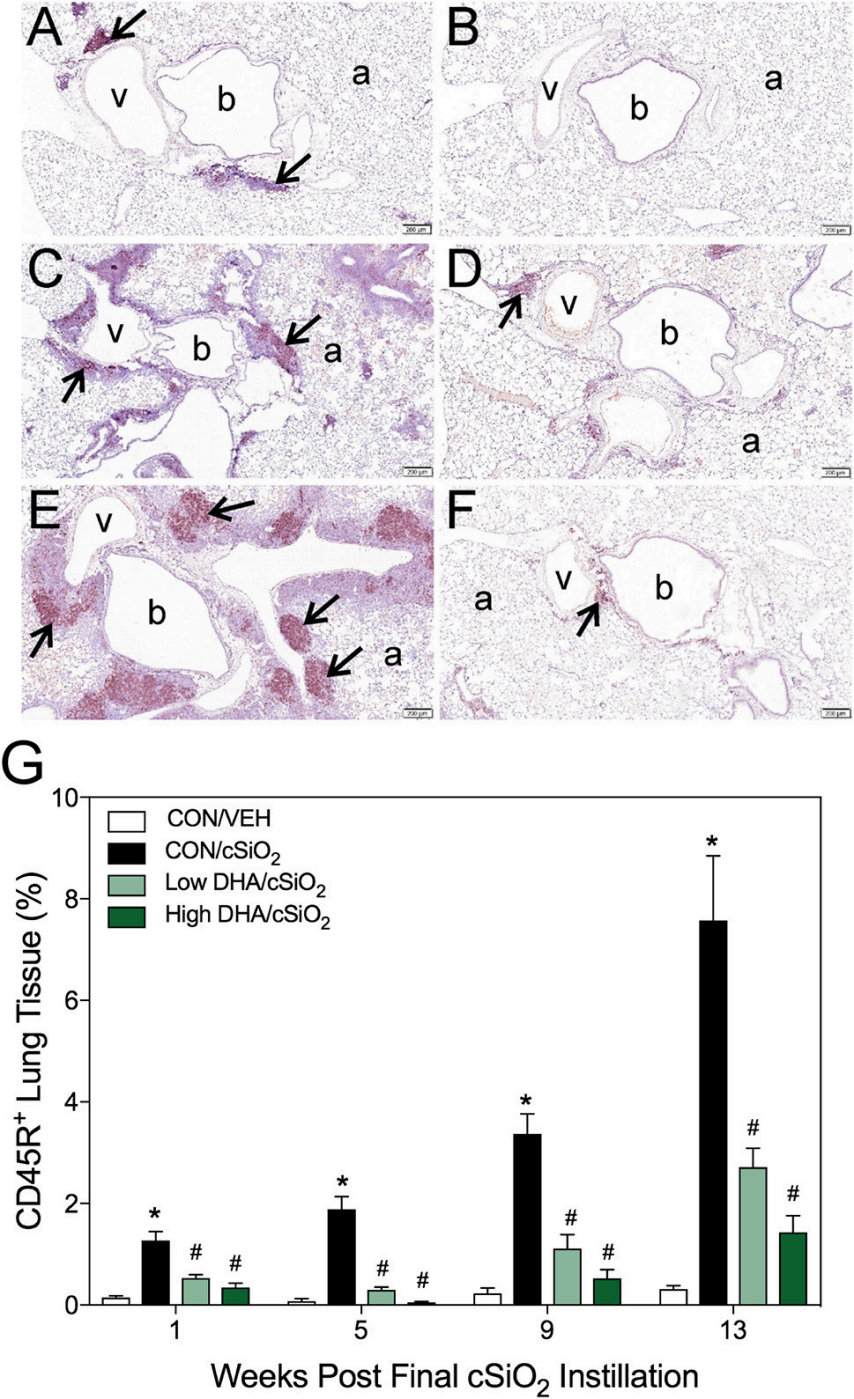
DHA consumption delays cSiO_2_-triggered B-cell appearance in lungs of NZBWF1 mice. Pulmonary densities of immunohistochemically labeled B-cells (CD45R^+^) in hematoxylin counterstained lung sections were immunohistochemically and morphometrically determined as described in Materials and Methods. Representative photomicrographs depict CD45R^+^ cells (reddish brown chromogen, indicated by arrows) in **(A)** CON/cSiO_2_ at 5 wk post-instillation (PI), **(C)** 9 wk PI, and **(E)** 13 wk PI; **(B)** CON/VEH at 13 wk PI; **(D)** Low DHA/cSiO_2_ at 13 wk PI; and **(F)** High DHA/cSiO_2_ at 13 wk PI. Abbreviations: a, alveolar parenchyma; b, bronchiolar airway; v, blood vessel. **(G)** Volume densities of CD45R^+^ cells are graphically expressed as means + SEM (*n* = 7–8 per group). Symbols: ^*^ indicates significantly different from CON/VEH group (*p* < 0.05); # indicates significantly different from CON/cSiO_2_ group (*p* < 0.05). Complete statistical analyses can be found in Supplementary Table 5.

**Figure 5:**
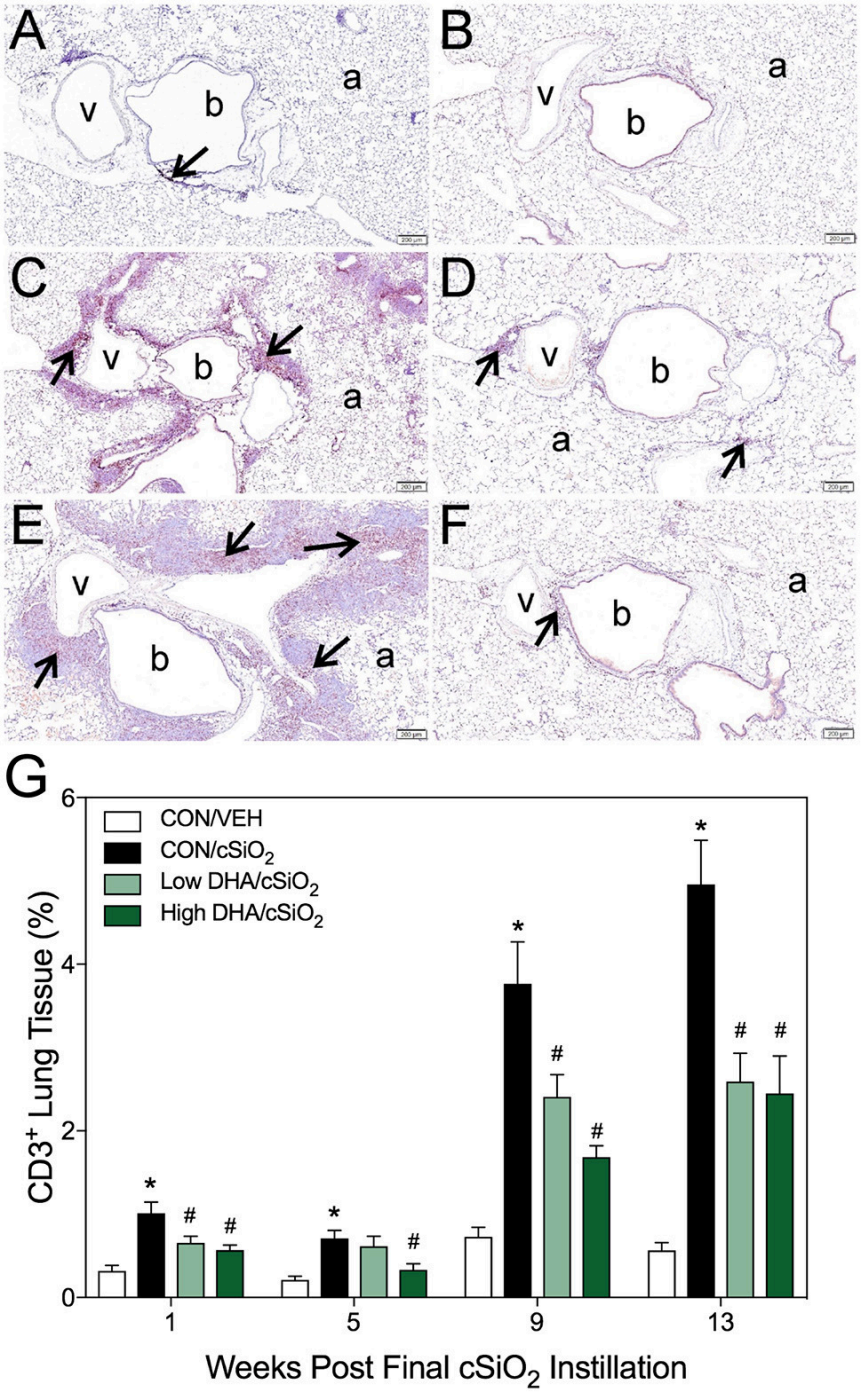
DHA consumption delays cSiO_2_-triggered T-cell appearance in lungs of NZBWF1 mice. Pulmonary densities of immunohistochemically labeled T-cells (CD3^+^), in hematoxylin counterstained lung sections were morphometrically determined as described in Materials and Methods. Representative photomicrographs depict CD3^+^ cells (reddish brown chromogen, indicated by arrows) in **(A)** CON/cSiO_2_ at 5 wk post-instillation (PI), **(C)** 9 wk PI, and **(E)** 13 wk PI; **(B)** CON/VEH at 13 wk PI; **(D)** Low DHA/cSiO_2_ at 13 wk PI; and **(F)** High DHA/cSiO_2_ at 13 wk PI. Abbreviations: a, alveolar parenchyma; b, bronchiolar airway; v, blood vessel. **(G)** Volume densities of CD3^+^ cells are graphically expressed as means + SEM (*n* = 7–8 per group). Symbols: ^*^ indicates significantly different from CON/VEH group (*p* < 0.05); # indicates significantly different from CON/cSiO_2_ group (*p* < 0.05). Complete statistical analyses can be found in Supplementary Table 5.

Morphometry was used on immunohistochemically stained slides from the lung to (1) quantify changes in B- and T-cells during cSiO_2_-induced pulmonary ectopic lymphoid neogenesis and (2) assess DHA's influence on these lymphocyte populations over time. cSiO_2_ elicited early and sustained CD45R^+^ B-cell and CD3^+^ T-cell elevation in lungs of mice fed CON diet at 1 and 5 wk PI (Figures 4A,G, 5A,G). B- and T- cells occurred mostly within diffuse interstitial infiltrates and did not appear to organize into distinct ELS at these time points. By 9 wk PI, cSiO_2_ instillation caused pronounced formation of focal B-cell aggregates interspersed with more diffuse T-cells in CON-fed mice, thus resembling ELS with functional germinal centers (Figures 4C,G, 5C,G). ELS in CON/cSiO_2_ group were dramatically expanded by 13 wk PI (Figures 4E,G, 5E,G) compared to VEH-treated mice fed control diet (Figures 4B, 5B). Notably, consumption of either Low DHA and High DHA diets was markedly effective at restricting cSiO_2_-induced B- and T-cell accumulation across all experimental time points (Figures 4D,F,G, 5D,F,G, Supplementary Table 5).

### DHA supplementation impedes cSiO_2_-induced formation ectopic germinal centers

**Figure 6:**
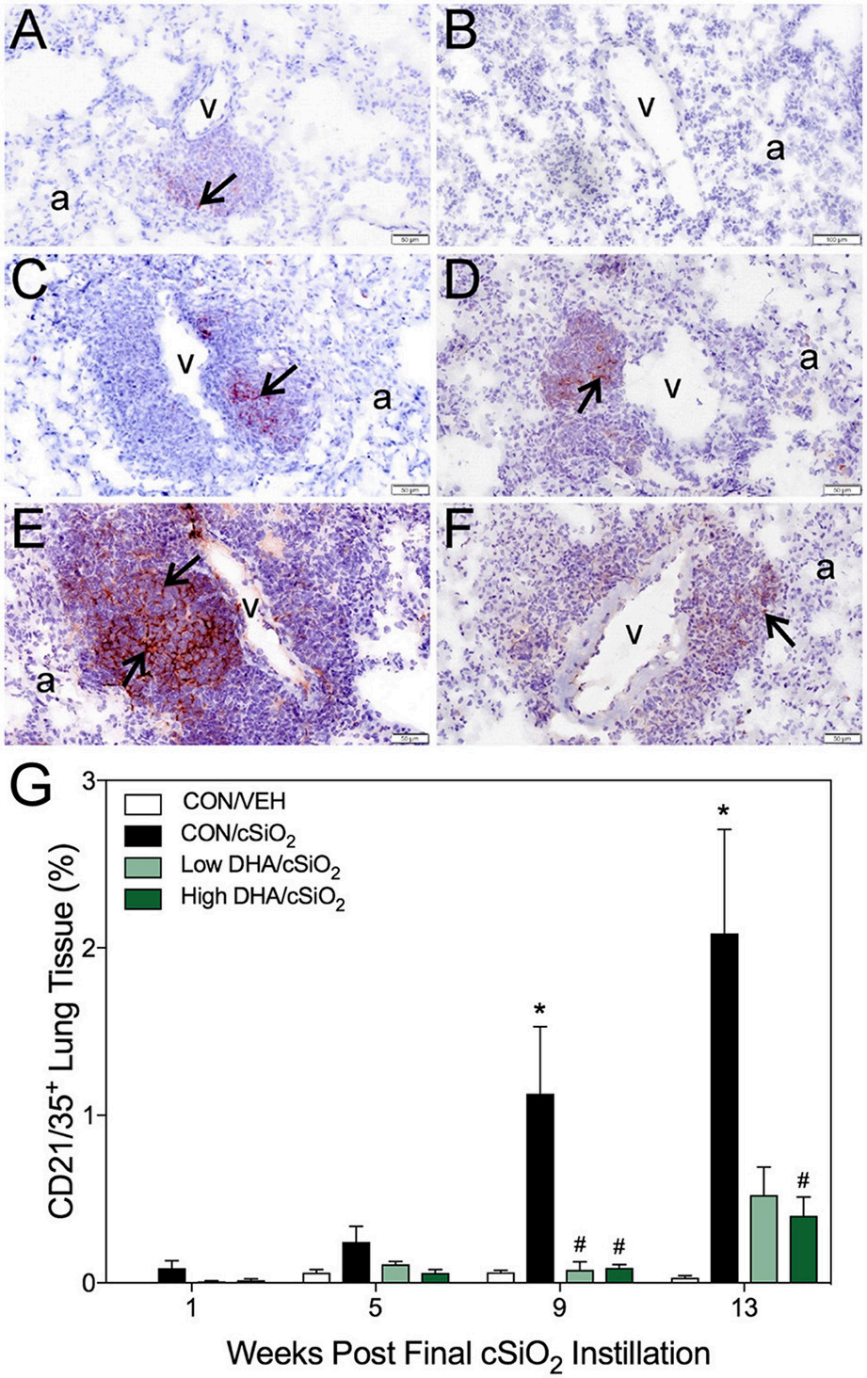
DHA consumption delays cSiO_2_-triggered follicular dendritic cell networking in lungs of NZBWF1 mice. Pulmonary densities of immunohistochemically labeled follicular dendritic cell (CD21/35^+^) networking in hematoxylin counterstained lung sections were morphometrically determined as described in Materials and Methods. Representative photomicrographs depict CD21/35^+^cells (reddish brown chromogen, indicated by arrows) in **(A)** CON/cSiO_2_ at 5 wk post-instillation (PI), **(C)** 9 wk PI, and **(E)** 13 wk PI; **(B)** CON/VEH at 13 wk PI; **(D)** Low DHA/cSiO_2_ at 13 wk PI; and **(F)** High DHA/cSiO_2_ at 13 wk PI. Abbreviations: a, alveolar parenchyma; v, blood vessel. **(G)** Volume densities of CD21/35^+^ cells are graphically expressed as means + SEM (*n* = 7–8 per group). Symbols: ^*^ indicates significantly different from CON/VEH group (*p* < 0.05); # indicates significantly different from CON/cSiO_2_ group (*p* < 0.05). Complete statistical analyses can be found in Supplementary Table 5.

**Figure 7:**
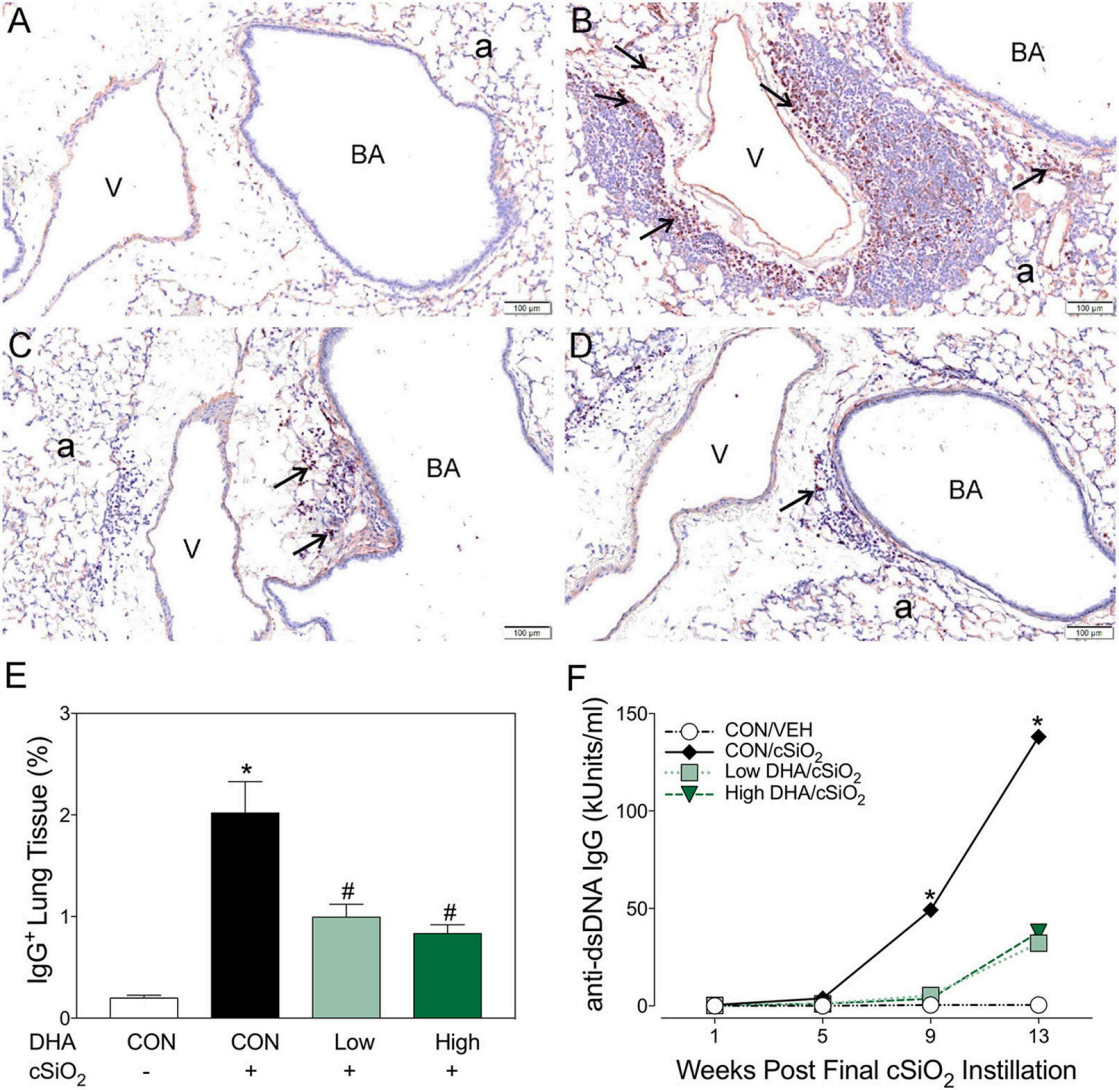
Dietary DHA suppresses cSiO_2_-triggered plasma cell influx in lungs and elevation of anti-dsDNA IgG in bronchoalveolar lavage fluid of NZBWF1 mice at 13 wk post-instillation. **(A–D)** Light photomicrographs of lung sections, immunohistochemically stained for IgG (dark reddish brown chromagen), from mice in the 13-wk post-exposure groups – **(A)** CON/VEH, **(B)** CON/cSiO_2_, **(C)** Low DHA/cSiO_2_ and **(D)** High DHA/cSiO_2_ mice. A marked infiltration of IgG^+^ plasma cells (arrows) was present in interstitial tissue surrounding blood vessels (v) and bronchiolar airways (BA) in the lungs of CON/cSiO_2_ mice **(B)**. Extracellular IgG was also conspicuous in alveolar airspaces (a) of these mice. Densities of IgG^+^ plasma cells and alveolar extracellular IgG were strikingly less in the lungs of both Low DHA/cSiO_2_ and High DHA/cSiO_2_ mice (**C** and **D**, respectively). Only a few widely scattered IgG^+^ plasma cells were occasionally present in the peribronchiolar and perivascular interstitial tissue of CON/VEH mice **(A)**. **(E)** Morphometrically determined volume densities of IgG^+^ plasma cells are graphically expressed as means + SEM (*n* = 7–8 per group). Symbols: ^*^ indicates significantly different from CON/VEH group (*p* < 0.05); # indicates significantly different from CON/cSiO_2_ group (*p* < 0.05). **(F)** Dietary DHA suppresses cSiO_2_ -induced elevation of anti-dsDNA IgG in bronchiolar lavage fluid (BALF). BALF was pooled for each experimental group and analyzed in duplicate. Data represent means ± SEM. ^*^ indicates significantly different from CON/VEH group (*p* < 0.05).

Immunohistochemistry for CD21^+^/CD35^+^ was used to quantify FDCs, markers of germinal centers, in ELS of CON/cSiO_2_ mice. FDC numbers were not affected by cSiO_2_ in lung sections from 1 or 5 wk PI (Figures 6A,G). However, by 9 and 13 wk PI, there was marked infiltration and networking of FDCs in CON/cSiO_2_ groups (Figures 6C,E,G) compared to corresponding CON/VEH mice (Figures 6B,G). FDCs were found predominately in the perivascular and peribronchial regions, which was consistent with the location of CD45R^+^ and CD3^+^ cell infiltrates. Remarkably, cSiO_2_-induced FDC accumulation was nearly completely ablated in mice consuming either DHA diets (Figures 6D,F,G, Supplementary Table 5). In concert with FDC findings, large aggregates (Figure 7B) of IgG^+^ plasma cells were present in the pulmonary perivascular and peribronchiolar interstitial tissue and were associated with ectopic lymphoid structures in CON/cSiO2 mice at wk 13. These were significantly diminished in the mice fed low and high DHA diets (Figures 7C,D,E). IgG^+^ plasma cells were few and widely scattered in these regions of lung in CON/VEH mice (Figures 7A,E). Accordingly, cSiO_2_ exposure elicited ectopic lymphoid germinal center development, but this response was blocked by inclusion of DHA in the diet.

### DHA suppresses induction of plasma anti-dsDNA autoantibodies by cSiO_2_

**Figure 8:**
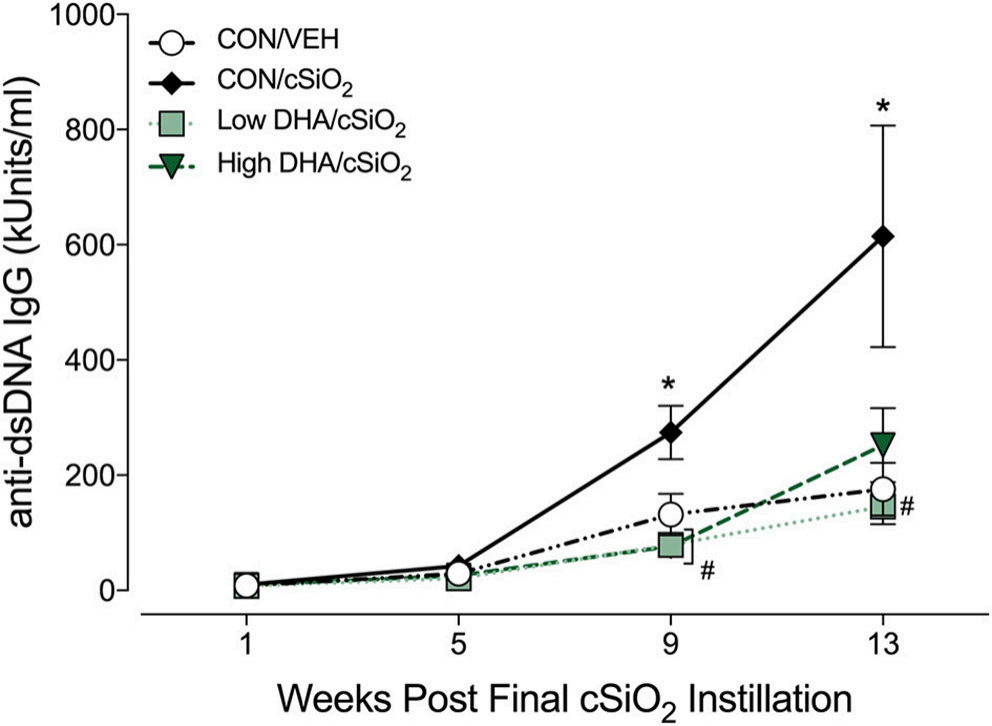
Dietary DHA suppresses *c*SiO_2_ -induced elevation anti-dsDNA IgG in plasma of NZBWF1 mice. Anti-dsDNA IgG in plasma of NZBWF1 mice was quantitated by ELISA. Data are means ± SEM (*n* = 7–8 per group). Symbols: ^*^ indicates significantly different from CON/VEH group (*p* < 0.05); # indicates significantly different from CON/cSiO_2_ group (*p* < 0.05). Complete statistical analyses can be found in Supplementary Table 5.

Robust anti-dsDNA IgG responses were observed in BALF (Figure 7F) and plasma (Figure 8, Supplementary Table 5) of cSiO_2_-instilled mice fed CON diet at 9 and 13 week PI compared to VEH-treated mice fed CON diet. Consumption of DHA suppressed these autoantibody responses. Thus, cSiO_2_-induced anti-dsDNA IgG responses in BALF and plasma and their suppression by DHA recapitulated the impact of these agents on ectopic germinal center development in the lung.

### Dietary DHA diminishes cSiO_2_-induced glomerulonephritis and B-cell infiltration in the kidney

**Figure 9:**
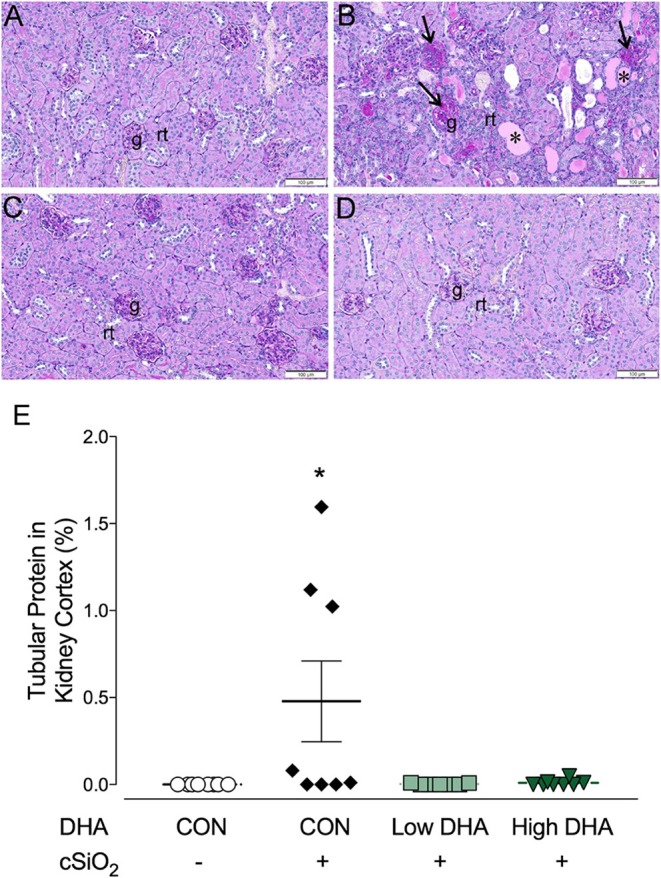
cSiO_2_ induced tubular proteinosis in kidneys of NZBWF1 mice at 13 wk PI. Light photomicrographs of kidney sections from NZBWF1 mice at 13 wk PI that were histochemically stained with Periodic acid-Schiff and hematoxylin (PASH) and morphometrically analyzed for tubular proteinosis. Significant lesions were detectable in 0/8, 5/8, 1/8, and 0/8 mice in the CON/VEH, CON/cSiO_2_, Low DHA/cSiO_2_, and High DHA/cSiO_2_ groups, respectively. Representative photomicrographs of the renal cortex in **(A)** CON/VEH; **(B)** CON/cSiO_2_; **(C)** Low DHA/cSiO_2_; and **(D)** CON/VEH at 13 wk PI; and **(D)** High DHA/cSiO_2_. Abbreviations: g, glomerulus; rt, renal tubules; ^*^, tubular protein; arrows, sclerotic glomerulus. **(E)** Morphometric assessment of tubular proteinosis. Data are means ± SEM (*n* = 8 per group). Symbol: ^*^ indicates significant difference (*p* < 0.05) between CON/VEH and CON/cSiO_2._ Complete statistical analyses can be found in Supplementary Table 6.

Kidneys were evaluated histologically for glomerulonephritis over the entire experimental time course. Significant lesions were apparent only at 13 wk PI with nephritis being detectable in 0, 5, 1, or 0 mice per group (*n* = 8) in the CON/VEH, CON/cSiO_2_, Low DHA/cSiO_2_, or High DHA/cSiO_2_ groups, respectively (Figures 9A–D). Tubular proteinosis was evident at 13 wk PI in the CON/cSiO_2_ group but not in the CON/VEH, Low DHA/cSiO_2_, or High DHA/cSiO_2_ groups (Figure 9E).

**Figure 10:**
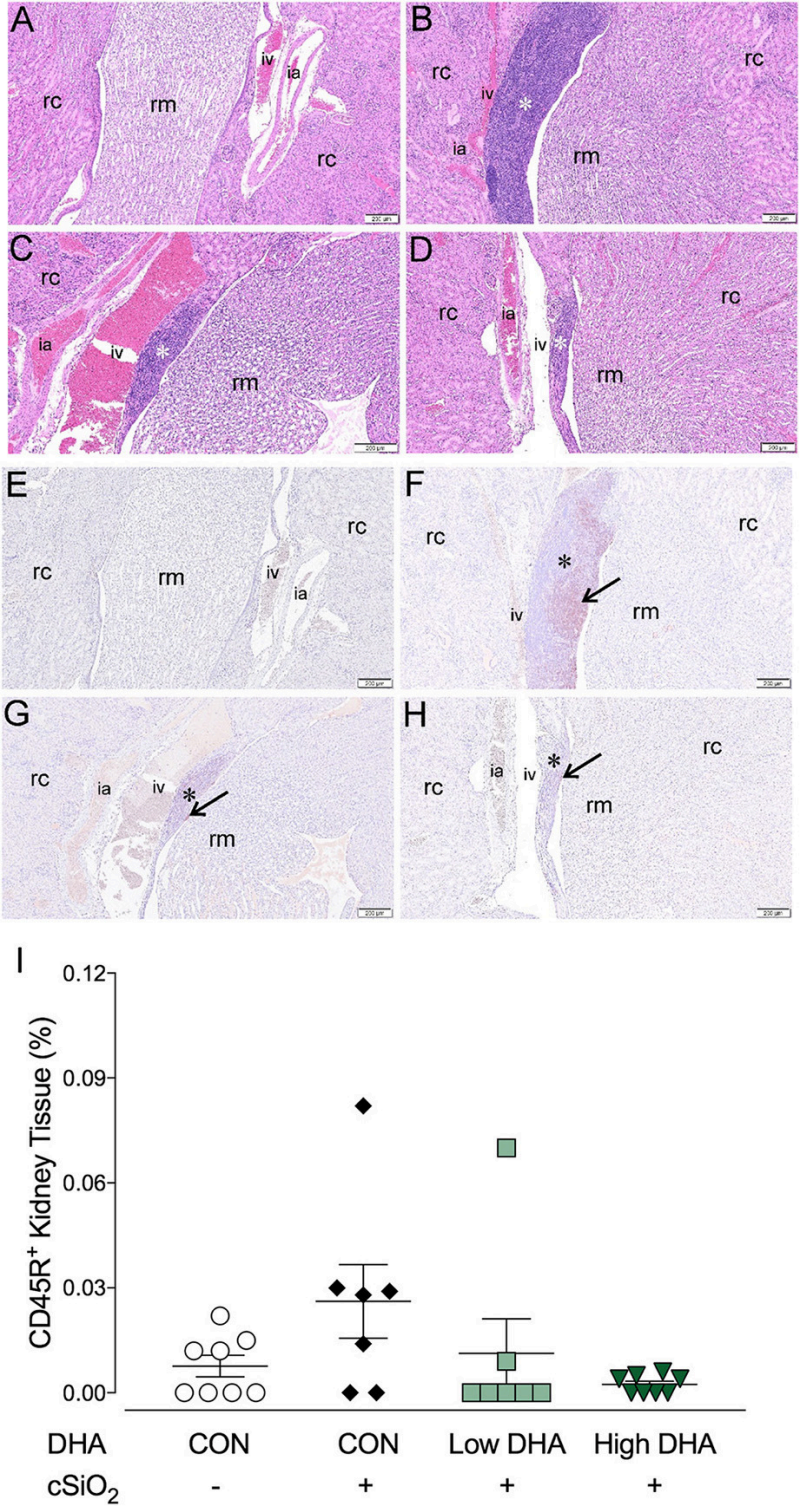
Lymphocytic and B-cell accumulation in kidneys of NZBWF1 mice 13 wk following cSiO_2_ exposure are prevented by dietary supplementation with DHA. Renal densities of lymphocyte and B-cell (CD45R^+^) were determined by routine light microscopic examination (H&E stained sections) and immunohistochemistry, respectively, as described in Materials and Methods. Representative light photomicrographs of H&E stained kidney sections depict **(A)** CON/VEH, **(B)** CON/cSiO_2_, **(C)** Low DHA/cSiO_2_; and **(D)** High DHA/cSiO_2_ at 13 wk PI. Light photomicrographs of CD45R^+^ cells (reddish brown chromogen, indicated by arrows) in lung sections from **(E)** CON/VEH, **(F)** CON/cSiO_2_, **(G)** Low DHA/cSiO_2_; and **(H)** High DHA/cSiO_2_ at 13 wk PI; and **(F)** High DHA/cSiO_2_ at 13 wk PI. Abbreviations: rc, renal cortex; rm, renal medulla; ia, intralobular artery; iv, intralobular vein. Asterisk indicates focal aggregates in the interstitium. **(I)** Volume densities of CD45R^+^ cells are graphically expressed as means ± SEM (*n* = 8 per group). No significant differences were identified for comparisons to CON/VEH or CON/cSiO_2_. Complete statistical analyses can be found in Supplementary Table 6.

B-cell infiltrates in the kidney have been previously associated with severity of lupus nephritis (58–60). Marked cell infiltration was evident in kidney tissues of mice in the CON/cSiO_2_ group at 13 wk PI compared to the other experimental groups. (Figures 10A–D). Immunohistochemistry revealed that in CON/cSiO_2_ mice kidneys, CD45R^+^ cells localized into distinct, focal aggregates in the interstitium surrounding interlobular arteries and veins in the renal cortex and near the renal pelvis (Figures 10E,F). A trend toward reduced cSiO_2_-triggered B-cell accumulation was evident in mice fed DHA (Figures 10G,H,I). Given that cSiO_2_ particles in this study are too large to diffuse into systemic circulation via the lung and are insoluble in water, it seems unlikely that these particles could be deposited in tissues distal to the lung. We did not observe cSiO_2_ particles in kidney by birefringent microscopy (unpublished observations) suggesting the improbability of direct deposition of cSiO_2_ particles in the kidney promoting CD45R^+^ B-cell infiltration.

## Discussion

Understanding how cSiO_2_ triggers autoimmunity in this preclinical model is important not only because exposure to this respirable particle has been linked to human autoimmune disease but also because this response serves as a model for agents that cause sterile inflammation. Inhalation of cSiO_2_ elicits a cascade that includes: (1) phagocytosis by alveolar macrophages, (2) lysosomal membrane permeabilization, (3) inflammasome activation, (4) release of inflammatory mediators, and (5) cell death and release of free cSiO_2_ particles (61–68). Since cSiO_2_ clearance from the lung is minimal (69), persistent repetition of this cascade evokes buildup of cell corpses, secondary necrosis, alarmin release, and self-antigen presentation that very likely culminate in early loss of tolerance and autoimmunity. Consistent with this scenario, we report here for the first time that cSiO_2_ instillation sequentially induces the accumulation of pulmonary B- and T-cell aggregates (1, 5 wk PI) → FDC networking and plasma cell accumulation in lung indicative of ectopic lymphoid germinal centers (9, 13 wk PI) → BALF and plasma anti-dsDNA IgG elevation (9, 13 wk PI) → glomerulonephritis with B-cell accumulation (13 wk PI). These observations provide unique insight into how respirable cSiO_2_ might induce initiation and potentially flaring in lupus and other human ADs.

While our data suggest that the lung is likely to be a rich source of anti-dsDNA antibodies in the plasma, it is also possible that these could also originate at other sites such as the spleen as a result of lymphocyte homing from the lung or stimulation of new germinal centers by IgG-immune complexes arising from the lung. However, we did not detect histological differences in splenic architecture in the 13 wk cohort that would be suggestive of changes in germinal center development between CON/VEH and CON/cSiO_2_ mice (data not shown).

Managing lupus involves reducing disease symptoms in newly diagnosed individuals and preventing progression of established tissue damage to organs such as kidney (70). Existing and emerging lupus therapies have multiple mechanisms of action, broadly encompassing non-specific immunosuppression, lymphocyte depletion, and neutralization of immune-stimulating cytokines and chemokines (70–73). These approaches have serious limitations including serious adverse effects, inability to reverse immune-mediated damage, and high costs (70, 74). Significantly, several studies have indicated that ELS function as a survival niches, potentially shielding autoreactive plasma cells from immunosuppressive therapies (75–77). Therefore, strategies are needed to block ELS development and limit their capacity to exacerbate lupus progression and severity.

Consumption of ω-3 PUFAs such as DHA represents an alternative and/or complementary approach to mitigate autoimmune disease and other chronic health conditions. Omega-3 PUFAs are the most widely consumed nutritional supplement after multivitamins, taken by ~30 million Americans (78, 79). Dietary ω-3 PUFA incorporation occurs in tissues and cells throughout the body, including immune cells. In general, ω-3 PUFAs are considered anti-inflammatory and promote resolution of inflammation, whereas ω-6 PUFAs are considered proinflammatory. Using FA content of erythrocytes, an established predictor of PUFA levels of other tissues in the body (57), we found here that dietary supplementation with DHA promoted incorporation of both DHA and another ω-3 PUFA, EPA, at the expense of the ω-6 PUFA ARA. Since the EPA content of the DHA diets was negligible, our findings suggest that there was enzymatic retroconversion of DHA to EPA as has been reported previously in mice fed DHA (80). A highly likely effect of increased ω-3 PUFA tissue content is skewing of immune cells toward less inflammatory phenotypes that dampen chronic inflammation and autoimmunity (81). In support of that contention, we show here that dietary DHA supplementation, at levels that mimic realistic human consumption, blocked or delayed (1) pulmonary B-cell, T-cell, and FDC networking; (2) plasma autoantibody increases; and (3) glomerulonephritis and B-cell infiltration in the kidneys of cSiO_2_-treated mice. Accordingly, our findings are consistent with the possibility that ω-3 PUFA supplementation might be practical strategy to intervene against an established environmental trigger of autoimmunity.

DHA and its retroconversion product EPA potentially quell cSiO_2_-triggered autoimmunity by altering bioactive lipid mediator production, intracellular signaling, transcription factor activity, gene expression, and membrane structure and function [reviewed in (40)]. Relative to bioactive lipid mediators, eicosanoids are a diverse category of metabolites that collectively include lipoxins, prostaglandins, and thromboxanes; these metabolites can influence both progression and resolution of inflammation. Eicosanoids are known to be induced in macrophages by cSiO_2_ (82–86) and are associated with human lupus. These bioregulators are derived from metabolism of free (non-esterified) FAs that are liberated from the plasma membrane by activated phospholipase A2 (PLA_2_). The free FAs serve as substrates for cyclooxygenase, lipoxygenase, or cytochrome P450 enzymes. Competition for these enzymes by liberated PUFAs results in different metabolite signatures that can potentiate or attenuate inflammation. Generally, metabolites derived from ω-6s like LA and ARA are considered proinflammatory, whereas ω-3s produce specialized pro-resolving metabolites (SPMs) that enhance resolution of tissue inflammation (87). DHA-derived SPMs include the D-series resolvins, protectins, and maresins. Of special relevance to the cSiO_2_ model, DHA and its SPMs have been shown to suppress inflammasome activation, prevent cell death, and enhance removal of cell corpses by efferocytosis (88–91). Toward this end, we are now focusing on how DHA and/or its SPMs influence alveolar macrophages responses to cSiO_2_ and how these events shape autoimmunity.

**Figure 11:**
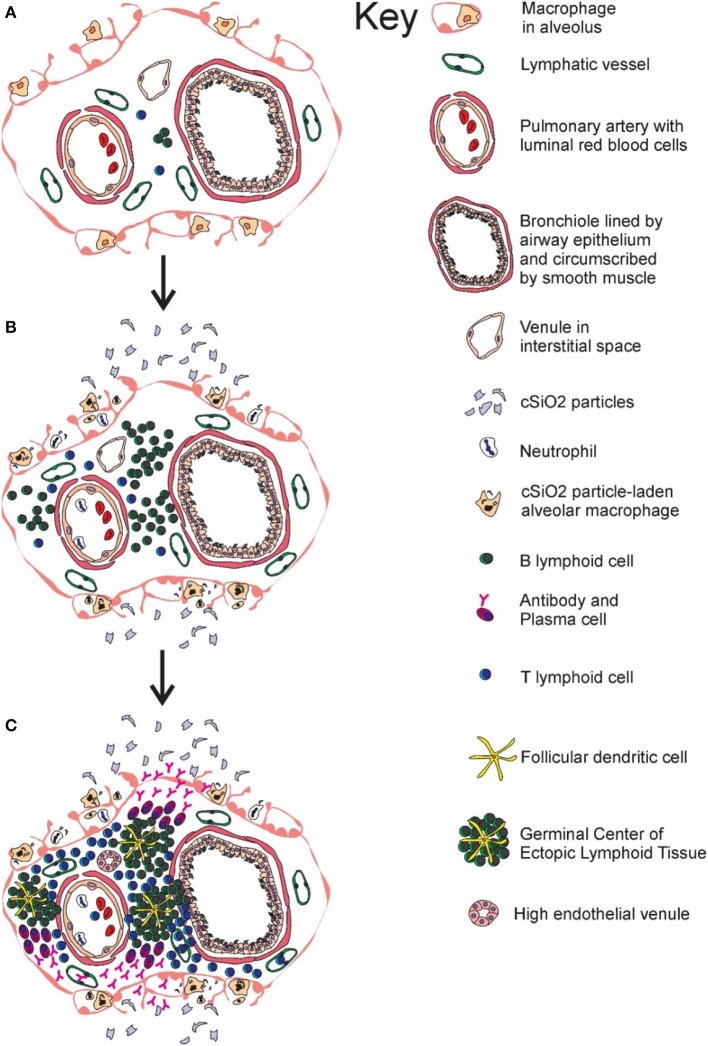
Temporal profile of cSiO_2_-triggered ectopic germinal center formation in the lung. Diagrammatic representation of lymphoid cell infiltration and development of ectopic lymphoid structures with germinal centers in the interstitial tissue surrounding a bronchiolar airway and adjacent blood vessel **(A)** prior to cSiO_2_ exposure, **(B)** at 1 and 5 wk PI, and **(C)** at 9 and 13 wk PI. Small accumulations of lymphoid cells are present in the perivascular and airway interstitial tissue at 1 wk PI with increasing numbers of lymphocytes (mainly B-cells) in the interstitial tissue at 5 wk PI. Distinct ectopic lymphoid structures characterized by follicular dendritic cell-laden germinal centers and numerous plasma cells were present by 9 and 13 wk after the final cSiO_2_ exposure.

Taken together, the results presented herein demonstrate that cSiO_2_ instillation in a lupus-prone mouse model sequentially induced *de novo* ectopic lymphoid germinal center formation in the lung (depicted in Figure 11), systemic autoantibody elevation, and glomerulonephritis. Furthermore, all of these responses were inhibited by inclusion of DHA in the diet. Upon considering the potential adverse outcomes of DHA and other ω-3 PUFAs, including those related to immune function, an expert committee of the European Food Safety Authority concluded that human intake up to 5 g/day is safe (92). Thus, the amounts of DHA used in this preclinical study, equivalent to 2 and 5 g/day human consumption, are consistent with realistic and safe doses in a clinical setting. Relative to human lupus, DHA supplementation may potentially aid two broad groups. One group is patients that are diagnosed with early-stage lupus where it could be used as a low-cost treatment, alone or as a complimentary approach to existing therapies. The second is individuals at increased risk for lupus who would benefit from a preventative measure with minimal side effects. This latter group could include asymptomatic individuals who have a close family member with lupus or workers occupationally exposed to respirable silica or related particles. Future preclinical studies using the cSiO_2_-triggered lupus-prone mouse models should focus on assessing the potential of DHA to serve as a therapeutic against established lupus and comparing the efficacy of DHA to established immunosuppressive agents used as the standard of care for individuals.

## Data availability

The raw data supporting the conclusions of this manuscript will be made available by the authors, without undue reservation, to any qualified researcher.

## Author contributions

MB: study design, animal study coordination, silica exposures, necropsy, sample handling, ELISA, immunohistochemistry, morphometry, data analyses, and drafting the manuscript; PA: study design, immunohistochemistry, morphometry, data analyses, and manuscript preparation; KG: animal study coordination, diet preparation, mouse feeding, silica exposures, sample handling, data analyses, manuscript preparation; AK: methods development, morphometry, data analyses; DJ-H, JW, and NL: coordination and conduct of necropsies, cytology, morphometry, histopathology; KW: sample analysis, data interpretation, manuscript preparation; CB: study design, methods development; AH: experimental design, data interpretation, manuscript writing, project funding; AB: data analysis, manuscript preparation; JH: study design, lung/kidney histopathology, morphometry, data analyses, manuscript preparation, project funding; JP: planning, coordination, oversight, manuscript preparation/submission, project funding.

### Conflict of interest statement

The authors declare that the research was conducted in the absence of any commercial or financial relationships that could be construed as a potential conflict of interest.

## Abbreviations

AD: autoimmune disease
ARA: arachidonic acid
BALF: bronchoalveolar lavage fluid
cSiO_2_: crystalline silica
CON: control
DHA: docosahexaenoic acid
ELS: ectopic lymphoid structure
EPA: eicosapentaenoic acid
FA: fatty acid
FDC: follicular dendritic cell
HRP: horseradish peroxidase
GLC: gas liquid chromatography
LA: linoleic acid
PBS: phosphate buffered saline
PI: post-instillation
PUFA: polyunsaturated fatty acid
RT: room temperature
SPMs: specific pro-resolving metabolite
VEH: vehicle.
