# Supplementary material for: Dietary Docosahexaenoic Acid Prevents Silica-Induced Development of Pulmonary Ectopic Germinal Centers and Glomerulonephritis in the Lupus-Prone NZBWF1 Mouse
Source: Front Immunol. 2018 Sep 12;9:2002. doi: 10.3389/fimmu.2018.02002 (PMC6143671; doi:10.3389/fimmu.2018.02002)
Supplement: Supplementary file 1 [file Table_1.PDF]

Supplementary Table 1. Fatty acid content of erythrocytes at 5 wk PI

		Experimental group			
		**CON/VEH**	**CON/cSiO$_2$**	**Low DHA/ cSiO$_2$**	**High DHA/ cSiO$_2$**
**Common Name**	**Chemical Formula**	*(% of total fatty acid)*			
Lauric Acid	C14:0	0.14 ± 0.02	0.15 ± 0.03	0.24 ± 0.04	0.31 ± 0.06
Palmitic Acid	C16:0	24.25 ± 0.59	23.96 ± 0.28	26.29 ± 0.56	28.44 ± 0.61
Palmitelaidic Acid	C16:1n7t	0.04 ± 0.01	0.04 ± 0.02	0.03 ± 0.01	0.04 ± 0.01
Palimitoleic Acid	C16:1n7	0.63 ± 0.20	0.60 ± 0.15	0.75 ± 0.14	0.76 ± 0.20
Stearic Acid	C18:0	15.36 ± 0.55	15.85 ± 0.57	14.84 ± 0.40	13.97 ± 0.17
Elaidic Acid	C18:1t	0.15 ± 0.01	0.13 ± 0.01	0.13 ± 0.01	0.13 ± 0.01
Oleic Acid	C18:1 ω-9	17.52 ± 0.61	17.64 ± 0.69	16.48 ± 0.70	15.91 ± 0.75
Linoelaidic Acid	C18:2 ω-6t	0.08 ± 0.1	0.07 ± 0.01	0.07 ± 0.01	0.06 ± 0.01
Linoleic Acid	C18:2 ω-6	9.09 ± 0.31	9.03 ± 0.37	12.34 ± 0.97	11.98 ± 0.85
Arachidic Acid	C20:0	0.13 ± 0.00	0.13 ± 0.01	0.12 ± 0.02	0.12 ± 0.01
Gamma-Linolenic Acid	C18:3 ω-6	0.06 ± 0.01	0.05 ± 0.01	0.05 ± 0.01	0.04 ± 0.01
Eicosenoic Acid	C20:1n9	0.37 ± 0.01	0.38 ± 0.02	0.27 ± 0.04	0.21 ± 0.03
Alpha-Linolenic Acid	C18:3 ω-3	0.04 ± 0.01	0.03 ± 0.01	0.05 ± 0.01	0.04 ± 0.01
Eicosadienoic Acid	C20:2 ω-6	0.24 ± 0.01	0.24 ± 0.01	0.23 ± 0.02	0.19 ± 0.02
Behenic Acid	C22:0	0.11 ± 0.02	0.12 ± 0.02	0.11 ± 0.03	0.11 ± 0.01
Dihomo-gamma-linolenic Acid	C20:3 ω-6	1.38 ± 0.04	1.39 ± 0.08	1.57 ± 0.09	1.11 ± 0.21
Arachidonic Acid	C20:4 ω-6	21.28 ± 0.62	21.14 ± 0.38	9.01 ± 0.74	3.89 ± 0.56
Lignoceric Acid	C24:0	0.22 ± 0.04	0.23 ± 0.04	0.24 ± 0.06	0.22 ± 0.01
Eicosapentaenoic Acid	C20:5 ω-3	0.23 ± 0.01	0.20 ± 0.01	2.60 ± 0.30	4.87 ± 0.19
Nervonic Acid	C24:1 ω-9	0.27 ± 0.06	0.29 ± 0.05	0.29 ± 0.06	0.23 ± 0.02
Adrenic Acid	C22:4 ω-6	2.13 ± 0.15	2.13± 0.02	0.34 ± 0.03	0.09 ± 0.02
Omega-6 Docosapentaenoic Acid	C22:5 ω-6	0.67 ± 0.04	0.75 ± 0.05	0.07 ± 0.01	0.04 ± 0.00
Omega-3 Docosapentaenoic Acid	C22:5 ω-3	0.49 ± 0. 03	0.45 ± 0.02	0.85 ± 0.06	0.88 ± 0.04
Docosahexaenoic Acid	C22:6 ω-3	5.14 ± 0.18	5.00 ± 0.31	13.05 ± 0.49	16.38 ± 0.67
	∑ SFA	40.21 ± 0.99	40.43 ± 0.51	41.83 ± 0.59	43.17 ± 0.48
	∑ MUFA	18.98 ± 0.82	19.09 ± 0.84	17.96 ± 0.82	17.38 ± 0.95
	∑PUFA (ω-3)	5.90 ± 0.18	5.68 ± 0.33	16.54 ± 0.73	22.17 ± 0.72
	∑ PUFA (ω-6)	34.93 ± 0.66	34.80 ± 0.51	23.67 ± 1.07	17.38 ± 0.61
