# Supplementary material for: Dietary Docosahexaenoic Acid Prevents Silica-Induced Development of Pulmonary Ectopic Germinal Centers and Glomerulonephritis in the Lupus-Prone NZBWF1 Mouse
Source: Front Immunol. 2018 Sep 12;9:2002. doi: 10.3389/fimmu.2018.02002 (PMC6143671; doi:10.3389/fimmu.2018.02002)
Supplement: Supplementary file 2 [file Table_2.PDF]

Supplementary Table 2. Fatty acid content of erythrocytes at 9 wk PI

		Experimental group			
		**CON/VEH**	**CON/cSiO$_2$**	**Low DHA/ cSiO$_2$**	**High DHA/ cSiO$_2$**
**Common Name**	**Chemical Formula**	*(% of total fatty acid)*			
Lauric Acid	C14:0	0.14 ± 0.04	0.15 ± 0.01	0.22 ± 0.06	0.25 ± 0.04
Palmitic Acid	C16:0	24.02 ± 0.42	23.71 ± 0.60	26.27 ± 0.48	27.73 ± 0.93
Palmitelaidic Acid	C16:1n7t	0.05 ± 0.01	0.04 ± 0.01	0.03 ± 0.01	0.03 ± 0.01
Palimitoleic Acid	C16:1n7	0.72 ± 0.34	0.64 ± 0.11	0.77 ± 0.31	0.76 ± 0.11
Stearic Acid	C18:0	14.92 ± 0.92	15.58 ± 0.73	14.38 ± 0.90	13.85 ± 0.35
Elaidic Acid	C18:1t	0.13 ± 0.01	0.14 ± 0.01	0.14 ± 0.03	0.11 ± 0.01
Oleic Acid	C18:1 ω-9	19.13 ± 1.51	18.40 ± 0.72	17.99 ± 0.88	17.81 ± 1.08
Linoelaidic Acid	C18:2 ω-6t	0.08 ± 0.01	0.08 ± 0.01	0.06 ± 0.02	0.05 ± 0.01
Linoleic Acid	C18:2 ω-6	7.88 ± 0.63	8.29 ± 0.59	10.42 ± 0.42	10.56 ± 0.46
Arachidic Acid	C20:0	0.11 ± 0.01	0.12 ± 0.01	0.11 ± 0.01	0.11 ± 0.02
Gamma-Linolenic Acid	C18:3 ω-6	0.06 ± 0.00	0.06 ± 0.01	0.05 ± 0.00	0.04 ± 0.01
Eicosenoic Acid	C20:1n9	0.37 ± 0.02	0.36 ± 0.02	0.32 ± 0.02	0.27 ± 0.03
Alpha-Linolenic Acid	C18:3 ω-3	0.03 ± 0.02	0.02 ± 0.01	0.03 ± 0.01	0.02 ± 0.01
Eicosadienoic Acid	C20:2 ω-6	0.20 ± 0.01	0.20 ± 0.01	0.24 ± 0.01	0.21 ± 0.01
Behenic Acid	C22:0	0.11 ± 0.04	0.08 ± 0.03	0.08 ± 0.02	0.08 ± 0.03
Dihomo-gamma-linolenic Acid	C20:3 ω-6	1.32 ± 0.11	1.26 ± 0.10	1.88 ± 0.13	1.50 ± 0.15
Arachidonic Acid	C20:4 ω-6	21.85 ± 1.17	22.16 ± 0.58	10.82 ± 0.63	4.90 ± 0.60
Lignoceric Acid	C24:0	0.19 ± 0.05	0.18 ± 0.05	0.18 ± 0.04	0.17 ± 0.05
Eicosapentaenoic Acid	C20:5 ω-3	0.10 ± 0.01	0.10 ± 0.01	2.01 ± 0.25	4.91 ± 0.49
Nervonic Acid	C24:1 ω-9	0.29 ± 0.07	0.30 ± 0.07	0.27 ± 0.05	0.26 ± 0.06
Adrenic Acid	C22:4 ω-6	2.25 ± 0.15	2.20± 0.09	0.47 ± 0.07	0.12 ± 0.02
Omega-6 Docosapentaenoic Acid	C22:5 ω-6	0.99 ± 0.12	0.92 ± 0.07	0.08 ± 0.02	0.03 ± 0.01
Omega-3 Docosapentaenoic Acid	C22:5 ω-3	0.33 ± 0. 03	0.31 ± 0.03	0.77 ± 0.04	0.96 ± 0.06
Docosahexaenoic Acid	C22:6 ω-3	4.71 ± 0.29	4.69 ± 0.16	12.41 ± 0.51	15.26 ± 0.84
	∑ SFA	39.52 ± 0.93	39.83 ± 0.89	41.24 ± 0.67	42.19 ± 0.94
	∑ MUFA	20.69 ± 1.81	19.88 ± 0.82	19.53 ± 1.17	19.24 ± 1.17
	∑PUFA (ω-3)	5.17 ± 0.28	5.12 ± 0.16	15.22 ± 0.68	21.15 ± 0.74
	∑ PUFA (ω-6)	34.63 ± 1.12	35.16 ± 0.77	24.01 ± 0.54	17.41 ± 0.70
