# Supplementary material for: Dietary Docosahexaenoic Acid Prevents Silica-Induced Development of Pulmonary Ectopic Germinal Centers and Glomerulonephritis in the Lupus-Prone NZBWF1 Mouse
Source: Front Immunol. 2018 Sep 12;9:2002. doi: 10.3389/fimmu.2018.02002 (PMC6143671; doi:10.3389/fimmu.2018.02002)
Supplement: Supplementary file 3 [file Table_3.PDF]

Supplementary Table 3. Fatty acid content of erythrocytes at 13 wk PI

		Experimental group			
		**CON/VEH**	**CON/cSiO$_2$**	**Low DHA/ cSiO$_2$**	**High DHA/ cSiO$_2$**
**Common Name**	**Chemical Formula**	*(% of total fatty acid)*			
Lauric Acid	C14:0	0.14 ± 0.06	0.11 ± 0.01	0.16 ± 0.02	0.17 ± 0.05
Palmitic Acid	C16:0	24.25 ± 0.59	25.06 ± 0.55	27.21 ± 0.80	28.69 ± 0.47
Palmitelaidic Acid	C16:1n7t	0.03 ± 0.00	0.03 ± 0.00	0.02 ± 0.00	0.02 ± 0.00
Palimitoleic Acid	C16:1n7	0.61 ± 0.31	0.41 ± 0.07	0.41 ± 0.37	0.52 ± 0.08
Stearic Acid	C18:0	15.45 ± 0.67	15.51 ± 0.89	14.22 ± 0.37	13.81 ± 0.64
Elaidic Acid	C18:1t	0.11 ± 0.01	0.12 ± 0.01	0.11 ± 0.01	0.10 ± 0.01
Oleic Acid	C18:1 ω-9	19.60 ± 1.62	19.01 ± 0.56	17.70 ± 0.51	17.41 ± 0.70
Linoelaidic Acid	C18:2 ω-6t	0.06 ± 0.02	0.06 ± 0.01	0.05 ± 0.01	0.05 ± 0.01
Linoleic Acid	C18:2 ω-6	8.33 ± 0.82	7.97 ± 0.76	9.99 ± 0.45	9.90 ± 0.70
Arachidic Acid	C20:0	0.12 ± 0.02	0.12 ± 0.02	0.11 ± 0.01	0.11 ± 0.02
Gamma-Linolenic Acid	C18:3 ω-6	0.06 ± 0.00	0.05 ± 0.01	0.05 ± 0.01	0.04 ± 0.01
Eicosenoic Acid	C20:1n9	0.40 ± 0.03	0.41 ± 0.04	0.33 ± 0.03	0.27 ± 0.04
Alpha-Linolenic Acid	C18:3 ω-3	0.02 ± 0.01	0.02 ± 0.00	0.02 ± 0.00	0.01 ± 0.00
Eicosadienoic Acid	C20:2 ω-6	0.20 ± 0.02	0.21 ± 0.03	0.24 ± 0.02	0.20 ± 0.02
Behenic Acid	C22:0	0.09 ± 0.06	0.07 ± 0.02	0.07 ± 0.01	0.05 ± 0.01
Dihomo-gamma-linolenic Acid	C20:3 ω-6	1.28 ± 0.16	1.16 ± 0.12	1.80 ± 0.27	1.47 ± 0.18
Arachidonic Acid	C20:4 ω-6	21.15 ± 1.27	21.37 ± 0.63	11.05 ± 0.78	5.06 ± 0.54
Lignoceric Acid	C24:0	0.11 ± 0.02	0.10 ± 0.03	0.12 ± 0.03	0.10 ± 0.02
Eicosapentaenoic Acid	C20:5 ω-3	0.08 ± 0.01	0.07 ± 0.01	1.98 ± 0.29	4.95 ± 0.51
Nervonic Acid	C24:1 ω-9	0.19 ± 0.03	0.19 ± 0.05	0.22 ± 0.05	0.20 ± 0.01
Adrenic Acid	C22:4 ω-6	2.36 ± 0.16	2.40 ± 0.32	0.54 ± 0.08	0.14 ± 0.02
Omega-6 Docosapentaenoic Acid	C22:5 ω-6	1.05 ± 0.09	1.18 ± 0.12	0.08 ± 0.01	0.03 ± 0.00
Omega-3 Docosapentaenoic Acid	C22:5 ω-3	0.27 ± 0. 03	0.25 ± 0.03	0.78 ± 0.04	1.01 ± 0.07
Docosahexaenoic Acid	C22:6 ω-3	4.03 ± 0.21	4.14 ± 0.22	12.75 ± 0.23	15.67 ± 0.70
	∑ SFA	40.17 ± 0.28	40.97 ± 0.78	41.89 ± 0.92	42.94 ± 0.42
	∑ MUFA	20.94 ± 1.91	20.17 ± 0.82	18.80 ± 0.54	18.53 ± 0.75
	∑PUFA (ω-3)	4.40 ± 0.23	4.48 ± 0.22	15.52 ± 0.43	21.64 ± 0.72
	∑ PUFA (ω-6)	34.49 ± 1.76	34.38 ± 0.89	23.79 ± 0.63	16.89 ± 1.05
