# Supplementary material for: Dietary Docosahexaenoic Acid Prevents Silica-Induced Development of Pulmonary Ectopic Germinal Centers and Glomerulonephritis in the Lupus-Prone NZBWF1 Mouse
Source: Front Immunol. 2018 Sep 12;9:2002. doi: 10.3389/fimmu.2018.02002 (PMC6143671; doi:10.3389/fimmu.2018.02002)
Supplement: Supplementary file 4 [file Table_4.PDF]

Supplemental Table 4. Statistical analyses of lung histopathology data

	ANOVA		Post-hoc test *p* values		
	F (DFn, DFd)	*p* value	CON/VEH vs. CON/cSiO2	CON/cSiO2 vs. Low DHA/cSiO2	CON/cSiO2 vs. High DHA/cSiO2
**Lymphoid aggregates**					
Interaction	F (9, 110) = 8.165	**<0.0001**			
Time point	F (3, 110) = 51.06	**<0.0001**			
Treatment	F (3, 110) = 66.54	**<0.0001**			
Post-hoc tests					
W1			0.0823	0.8725	0.3088
W5			**0.0038**	0.5093	0.0725
W9			**<0.0001**	0.4724	**0.0003**
W13			**<0.0001**	**0.0495**	**0.0038**
**Ectopic lymphoid tissues**					
Interaction	F (9, 110) = 17.16	**<0.0001**			
Time point	F (3, 110) = 92.37	**<0.0001**			
Treatment	F (3, 110) = 61.45	**<0.0001**			
Post-hoc tests					
W1			0.1261	0.1261	0.1261
W5			0.5576	0.5127	0.5576
W9			**<0.0001**	0.0722	**0.0005**
W13			**<0.0001**	0.0581	**0.0065**
**Alveolar proteinosis**					
Interaction	F (9, 110) = 29.98	**<0.0001**			
Time point	F (3, 110) = 265.2	**<0.0001**			
Treatment	F (3, 110) = 466	**<0.0001**			
Post-hoc tests					
W1			**<0.0001**	1.0000	0.7746
W5			**0.0003**	1.0000	1.0000
W9			**<0.0001**	1.0000	1.0000
W13			**<0.0001**	1.0000	1.0000
**Alveolitis**					
Interaction	F (9, 110) = 9.831	**<0.0001**			
Time point	F (3, 110) = 61.7	**<0.0001**			
Treatment	F (3, 110) = 146.3	**<0.0001**			
Post-hoc tests					
W1			**0.0002**	1.0000	**0.0367**
W5			**<0.0001**	1.0000	1.0000
W9			**<0.0001**	0.7210	**0.0221**
W13			**<0.0001**	0.4918	0.1104
**Type 2 cell hyperplasia**					
Interaction	F (9, 110) = 8.872	**<0.0001**			
Time piont	F (3, 110) = 74.47	**<0.0001**			
Treatment	F (3, 110) = 16.56	**<0.0001**			
Post-hoc tests					
W1			**n/a**	**n/a**	**n/a**
W5			**n/a**	**n/a**	**n/a**
W9			**0.0411**	1.0000	1.0000
W13			**<0.0001**	1.0000	0.7745
**Mucous cell metaplasia**					
Interaction	F (9, 110) = 1.334	0.2278			
Time point	F (3, 110) = 7.232	**0.0002**			
Treatment	F (3, 110) = 1.3	0.2780			
Post-hoc tests					
W1			**n/a**	**n/a**	**n/a**
W5			**n/a**	**n/a**	**n/a**
W9			**n/a**	**n/a**	**n/a**
W13			0.6221	1.0000	1.0000

Note: Data were analyzed by nonparametric Kruskal-Wallis ANOVA on ranks with Dunn's post-hoc tests for multiple comparisons.
