# Supplementary material for: Dietary Docosahexaenoic Acid Prevents Silica-Induced Development of Pulmonary Ectopic Germinal Centers and Glomerulonephritis in the Lupus-Prone NZBWF1 Mouse
Source: Front Immunol. 2018 Sep 12;9:2002. doi: 10.3389/fimmu.2018.02002 (PMC6143671; doi:10.3389/fimmu.2018.02002)
Supplement: Supplementary file 5 [file Table_5.PDF]

Supplemental Table 5. Statistical analyses of lung immunohistochemistry, BALF and anti-dsDNA IgG data

	ANOVA		Post-hoc test *p* values		
	F (DFn, DFd)	*p* value	CON/VEH vs. CON/cSiO2	CON/cSiO2 vs. Low DHA/cSiO2	CON/cSiO2 vs. High DHA/cSiO2
**CD3**					
Interaction	F (9, 110) = 9.229	**<0.0001**			
Time point	F (3, 110) = 71.05	**<0.0001**			
Treatment	F (3, 110) = 47.33	**<0.0001**			
Post-hoc tests					
W1			**<0.0001**	**0.0087**	**0.0030**
W5			**0.0022**	0.4694	**0.0118**
W9			**<0.0001**	**0.0037**	**<0.0001**
W13			**<0.0001**	**0.0002**	**0.0002**
**CD45R**					
Interaction	F (9, 106) = 11.16	**<0.0001**			
Time point	F (3, 106) = 42.07	**<0.0001**			
Treatment	F (3, 106) = 68.92	**<0.0001**			
Post-hoc tests					
W1			**<0.0001**	**0.0048**	**<0.0001**
W5			**<0.0001**	**<0.0001**	**<0.0001**
W9			**<0.0001**	**0.0078**	**<0.0001**
W13			**<0.0001**	**0.0026**	**<0.0001**
**CD21/35**					
Interaction	F (9, 99) = 5.171	**<0.0001**			
Time point	F (3, 99) = 13.1	**<0.0001**			
Treatment	F (3, 99) = 17.89	**<0.0001**			
Post-hoc tests					
W1			0.0539	0.0966	0.2566
W5			0.4851	1.0000	0.3890
W9			**<0.0001**	**<0.0001**	**0.0001**
W13			**<0.0001**	0.0922	**0.0246**
**BALF total cells**					
Interaction	F (9, 108) = 27.45	**<0.0001**			
Time point	F (3, 108) = 74.27	**<0.0001**			
Treatment	F (3, 108) = 130.4	**<0.0001**			
Post-hoc tests					
W1			**0.0001**	1.0000	**0.0315**
W5			**<0.0001**	0.3367	0.0850
W9			**<0.0001**	**0.0150**	**<0.0001**
W13			**<0.0001**	**0.0144**	**<0.0001**
**anti-dsDNA IgG**					
Interaction	F (9, 100) = 3.057	**0.0028**			
Time point	F (3, 100) = 19.27	**<0.0001**			
Treatment	F (3, 100) = 7.256	**0.0002**			
Post-hoc tests					
W1			0.6857	0.5417	0.9731
W5			0.4356	0.0884	0.2608
W9			**0.0099**	**0.0007**	**0.0006**
W13			**0.0369**	**0.0317**	0.1017

Note: Data were analyzed by ANOVA with Sidak's post-hoc tests for multiple comparisons.
