# Supplementary material for: Dietary Docosahexaenoic Acid Prevents Silica-Induced Development of Pulmonary Ectopic Germinal Centers and Glomerulonephritis in the Lupus-Prone NZBWF1 Mouse
Source: Front Immunol. 2018 Sep 12;9:2002. doi: 10.3389/fimmu.2018.02002 (PMC6143671; doi:10.3389/fimmu.2018.02002)
Supplement: Supplementary file 6 [file Table_6.PDF]

Supplemental Table 6. Statistical analyses of kidney parameters

	ANOVA		Post-hoc test *p* values		
	Test statistic	*p* value	CON/VEH vs. CON/cSiO2	CON/cSiO2 vs. Low DHA/cSiO2	CON/cSiO2 vs. High DHA/cSiO2
Tubular protein	H = 8.957	**0.0299**	**0.0107**	0.1451	0.4354
CD45R+ cells	F = 2.125	0.1214	0.1353	0.1543	0.0663

Note: Tubular protein data were analyzed by nonparametric Kruskal-Wallis ANOVA on ranks with Dunn's post-hoc tests for multiple comparisons. CD45R+ data were analyzed by ANOVA with Sidak's post-hoc tests for multiple comparisons.
